# Metal-Mediated Halogen Exchange in Aryl and Vinyl Halides: A Review

**DOI:** 10.3389/fchem.2018.00114

**Published:** 2018-04-26

**Authors:** Gwilherm Evano, Antoine Nitelet, Pierre Thilmany, Damien F. Dewez

**Affiliations:** Laboratoire de Chimie Organique, Service de Chimie et PhysicoChimie Organiques, Université libre de Bruxelles, Brussels, Belgium

**Keywords:** halogen exchange, finkelstein, aryl halides, vinyl halides, aryl fluorides, catalysis

## Abstract

Halogenated arenes and alkenes are of prime importance in many areas of science, especially in the pharmaceutical, agrochemical, and chemical industries. While the simplest ones are commercially available, some of them are still hardly accessible depending on their substitution patterns and the nature of the halogen atom. Reactions enabling the selective and efficient replacement of the halogen atom of an aryl or alkenyl halide by another one, lighter, or heavier, are therefore of major importance since they can be used for example to turn a less reactive aryl/alkenyl chloride into the more reactive iodinated derivatives or, in a reversed sense, to block an undesired reactivity, for late-stage modifications or for the introduction of a radionuclide. If some halogen exchange reactions are possible with activated substrates, they usually require catalysis with metal complexes. Remarkably efficient processes have been developed for metal-mediated halogen exchange in aryl and vinyl halides: they are overviewed, in a comprehensive manner, in this review article.

## Introduction

Aryl and vinyl halides are among the most important building blocks in organic chemistry. They are indeed starting materials for a range of metal-mediated cross-coupling reactions such as, just to mention a few, the Heck, Stille, Suzuki-Miyaura, Sonogashira-Linstrumelle (Tsuji, 2005), or Ullmann-Goldberg (Evano and Blanchard, 2013) reactions that are used on a daily basis in most areas of science where there is a strong need for small molecules as well as for the industrial production of a number of molecules that have a significant impact on our daily lives (Torborg and Beller, 2009; Dumrath et al., 2013; Evano and Blanchard, 2013).

In addition to their usefulness as reagents involved in an ever-growing number of transformations, they are also at the core structure of many natural products, drugs and agrochemicals. Moreover, they are also crucial to various imaging techniques such as positron-emission (PET) or single-photon emission computed (SPECT) tomographies for which radiotracers embedded with labeled aryl fluorides (^18^F) and iodides (^123^I) are important.

In all these areas, the nature of the halogen is of dramatic importance: aryl/vinyl iodides are typically more reactive in cross-coupling reactions than the corresponding bromides or chlorides and the halogen atom used in molecules from medicinal chemistry or agrochemistry and for imaging techniques is also crucial. From practical and economical points of view, the availability of aryl and vinyl halides typically follows the following order: Cl > Br > I > F while their cost is inversely proportional. From a purely synthetic viewpoint, the ease with which the electrophilic halogenation of arenes is performed increases when moving down the halogen column in the periodic table and alkenyl halides are much easier to prepare than their lighter counterparts. As for ^18^F- or ^123^I- labeled molecules, the only parameters that matter are the rate and efficiency of the incorporation of the radionuclides into an arene or an alkene considering the short half-lives of these compounds.

An obvious conclusion can be easily drawn from these rather trivial facts and considerations: reactions enabling the selective and efficient replacement of one halogen atom into an aryl or vinyl halide by another one, lighter, or heavier, are of major importance. Indeed, they for example enable turning a less reactive aryl chloride into a more reactive aryl iodide, they can be used, in a reversed sense, to block an undesired reactivity, they can be used for late-stage modification of a drug or for the introduction of a radionuclide.

**Figure 1:**
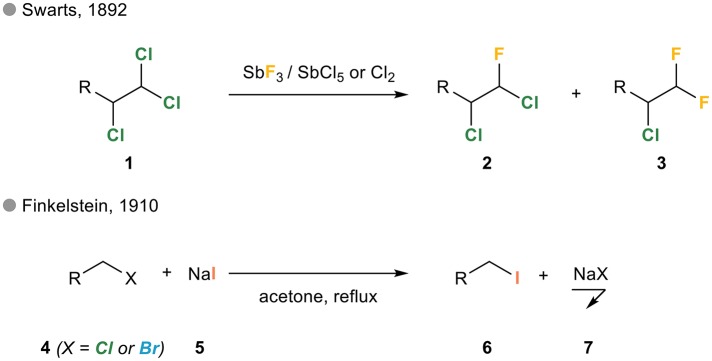
Halogen exchange in alkyl halides: the Swarts and Finkelstein reactions.

These “halogen exchange reactions” have been known for more than a century in the aliphatic series. The first example actually dates back to 1892 when Belgian chemist Frédéric Swarts reported the substitution of one or more chloride(s) in polychlorinated alkanes **1** by fluorides upon treatment with antimony trifluoride in the presence of antimony pentachloride or chlorine (Figure 1; Swarts, 1892a,b). Eighteen years later, German chemist Hans Finkelstein reported what would become the Finkelstein reaction, i.e., the reaction of an alkyl bromide or chloride **4** and potassium iodide **5** in refluxing acetone yielding the corresponding iodinated alkane upon precipitation of sodium chloride or bromide **7** (Finkelstein, 1910).

These reactions, which proceed by nucleophilic substitution, of course cannot take place with aryl or vinyl halides, except in peculiar cases where an aromatic nucleophilic substitution or an addition/elimination occurs. The use of a metal catalyst however enables these aromatic and vinylic halogen exchange reactions: due to their strong potential, such processes have been extensively studied, which recently led to the development of efficient and broadly applicable catalytic systems that smoothly facilitate a range of halogen exchange reactions. They will be overviewed in this review article in which they have been classified according to the type of substrate utilized (aromatic vs. vinylic), the direction in which the halogen exchange operates (from lighter to heavier halogen atoms or vice versa), and the nature of the catalyst used; halogen exchange reactions enabling the introduction of a fluorine atom at a sp^2^ carbon atom and synthetic applications of these processes will be treated separately. As an important note, this review does not intend to be exhaustive but will rather focus on the most significant developments: the reader should refer to previous review articles on this topic. (Sheppard, 2009; Casitas and Ribas, 2013a). Moreover, representative examples have been incorporated for all procedures which provide full conversions and whose generality and applicability was clearly demonstrated to further highlight their synthetic usefulness.

**Figure 2:**
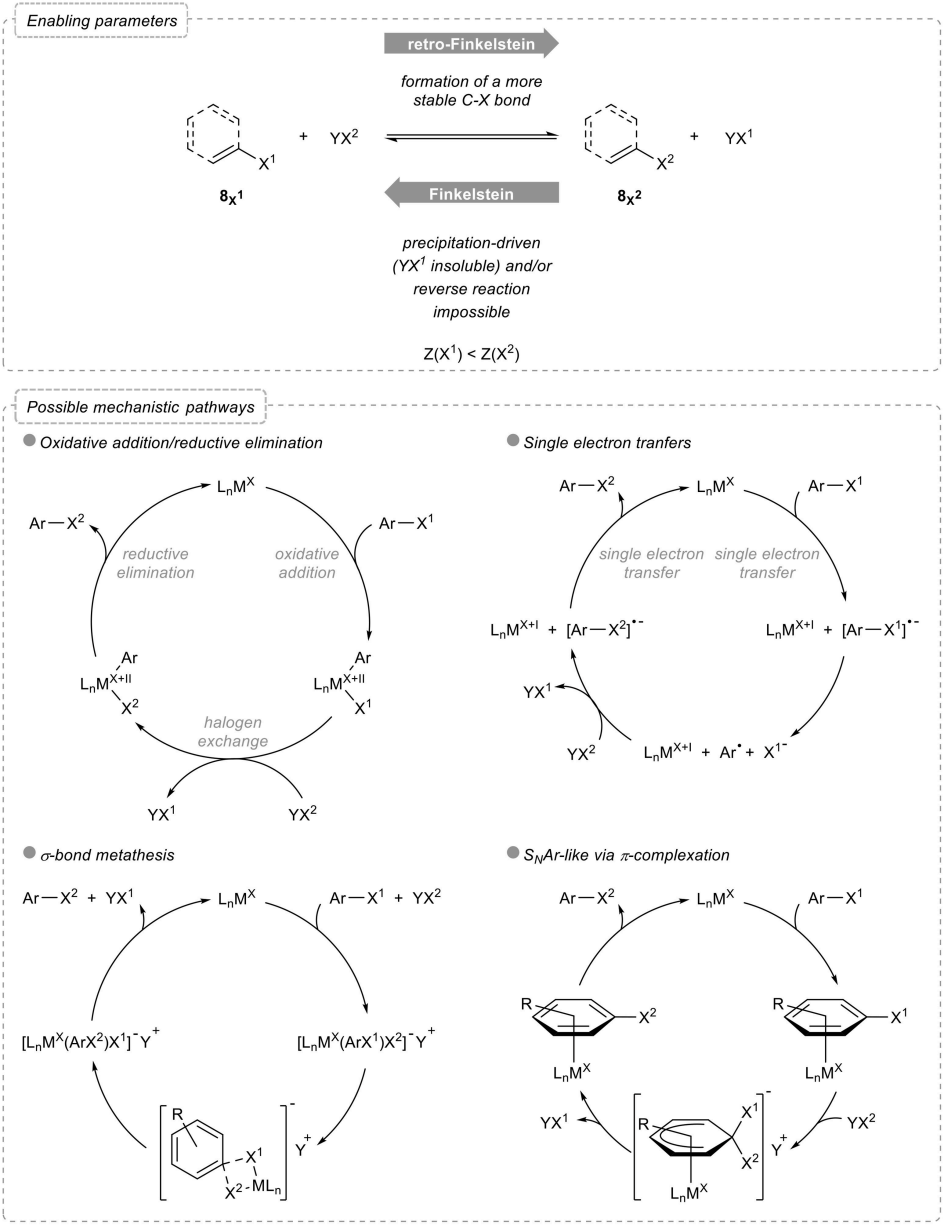
Halogen exchange in aryl and vinyl halides: enabling parameters and possible mechanistic pathways.

Before starting with the overview of these reactions, some useful and general considerations should be examined. Indeed, a halogen exchange reaction being used to transform an organic halide into another one, the reverse reaction should be feasible and potentially lead to an equilibrium. Various parameters therefore need to be taken into account to perform such a reaction (Figure 2):

- Relative stabilities: exchanging a halogen atom by a lighter one, which actually corresponds to a retro-Finkelstein reaction, yields to a more stable product, as evidenced by the relative bond dissociation energies: $D_{298}^{{}^{\circ}}$(C-F) = 525 kJ mol^−1^, $D_{298}^{{}^{\circ}}$(C-Cl) = 400 kJ mol^−1^, $D_{298}^{{}^{\circ}}$(C-Br) = 336 kJ mol^−1^, and $D_{298}^{{}^{\circ}}$(C-I) = 272 kJ mol^−1^ (Haynes, 2016).
- Shifting of chemical equilibrium: in the reverse sense, the Finkelstein reaction actually yields to the formation of a less stable product. Although this could be somehow potentially balanced by the greater stability of YX^1^ compared to that of the starting halogenating agent YX^2^, differences in their relative stabilities are generally much less pronounced, due to their ionic nature, than in the aryl halides series [one can compare for example $D_{298}^{{}^{\circ}}$(Na-Cl) = 20 kJ mol^−1^, $D_{298}^{{}^{\circ}}$(Na-Br) = 58 kJ mol^−1^ and $D_{298}^{{}^{\circ}}$(Na-I) = 65 kJ mol^−1^ (Haynes, 2016)]. The equilibrium is therefore often shifted by precipitation of the halogen salt formed in the process and in this case, both the counterion and the solvent have a crucial role.
- Reversibility of the process: aromatic and vinylic Finkelstein reactions enabling the transformation of aryl and vinyl halides $8_{{\text{X}}^{2}}$ to their heavier homologs $8_{{\text{X}}^{1}}$ typically rely on the use of transition metals which activate the carbon-halogen bond of the starting organic halide via, for example, oxidative addition. Both the feasibility and the rate of this activation step strongly depend on the nature of the halogen atoms and the process might therefore not be reversible.

As for the possible mechanistic pathways, most of them involving metal catalysts or mediators, four main alternatives can be considered. The first and probably most classical one involves an oxidative addition followed by halogen exchange and reductive elimination, the rate and equilibrium constants for both the oxidative addition and reductive elimination being the main factors to be considered for the overall halogen exchange to proceed. Alternatively, single electron transfers can be operative, the metal in this case reducing the starting aryl halide, which triggers the cleavage of the carbon-halogen bond: further recombination of the aryl radical with the other halide and concomitant reduction then closes the catalytic cycle and account for the exchange of halogens. Less common pathways involve either σ-bond metathesis via a four-membered intermediate or transition state or π-complexation of the metal to the arene.

Based on these simple enabling parameters and basic mechanistic considerations, various processes have been designed and developed, mostly based on the use of transition metal complexes, to perform the halogen exchange reaction in aryl and vinyl halides. They will be overviewed in this review article, starting with aromatic Finkelstein reactions.

## Halogen exchange in aryl halides

### Aromatic finkelstein reactions

As mentioned above, aromatic Finkelstein reactions correspond to the formal substitution of a halogen atom by a heavier one in aryl halides. Among all metal-based catalytic systems reported to promote such reactions, nickel complexes have been widely investigated and were actually the first used for the catalysis of aromatic Finkelstein reactions.

#### Catalyzed/mediated by nickel complexes

**Figure 3:**
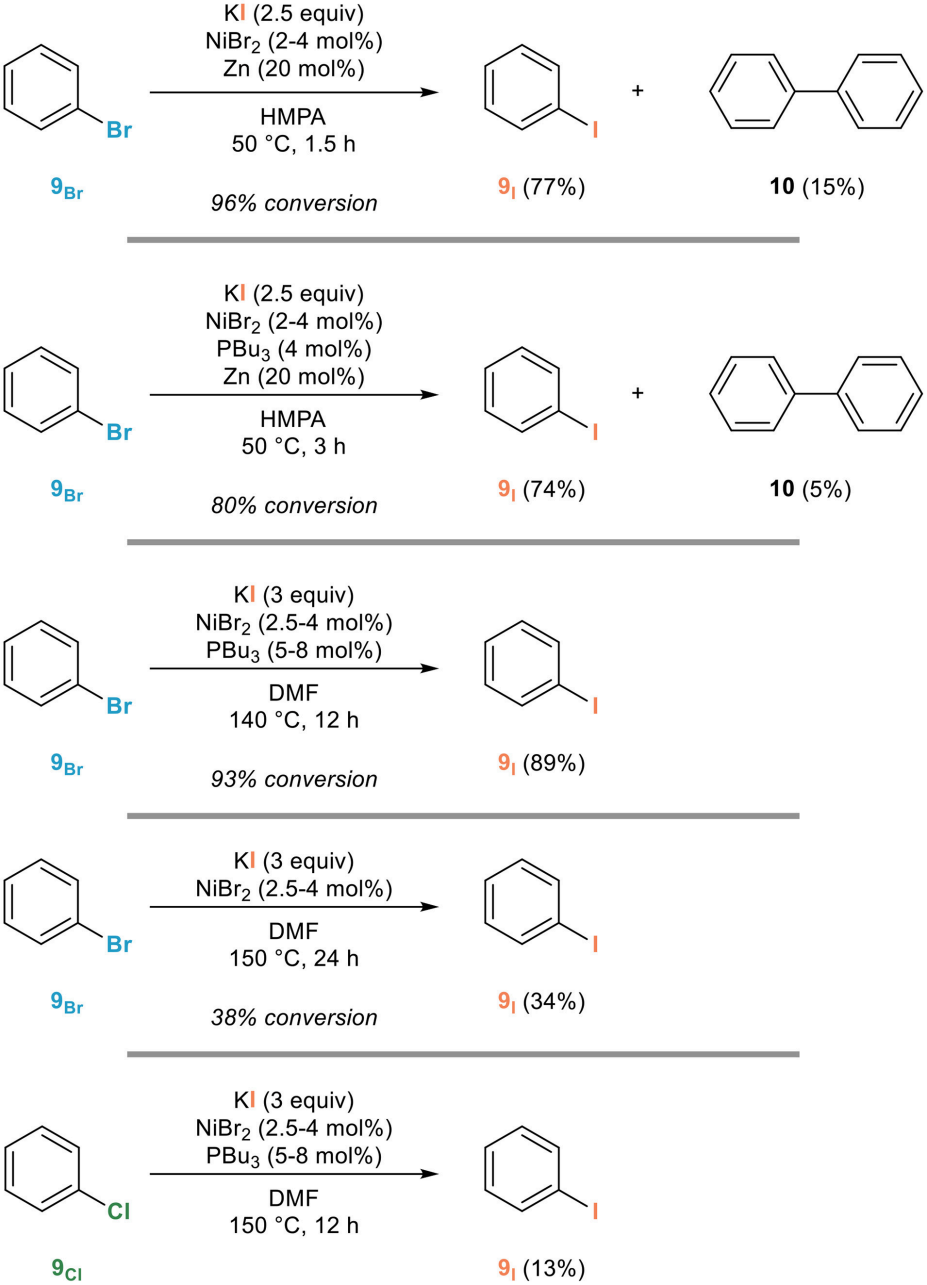
Influence of additives in the nickel-catalyzed aromatic Finkelstein reaction.

One of the first examples of an aromatic Finkelstein reaction was actually reported in the late 1970s by the Takagi group (Takagi et al., 1978b). This remarkable study reveals the side reactions that are to be avoided—notably the homocoupling to biaryls and the reduction—for the development of an efficient process. An evaluation of the effect of various additives led to the development of a catalytic system based on the combination of nickel(II) bromide in the presence of tributylphosphine: this donor ligand suppresses the competing dimerization to **10** at the expense of the rate of the reaction since it had to be performed at 140°C in HMPA instead of 50°C. At such an elevated temperature, no reducing agent was required while zinc was requested when performing the reaction at 50°C. Representative examples for the conversion of bromobenzene **9**_Br_ to iodobenzene **9**_I_ shown in Figure 3 are illustrative of the influence of these parameters. As important notes, the use of chlorobenzene **9**_Cl_ in place of bromobenzene led to a much less efficient reaction and the mechanism proposed involves the generation of a nickel(0) catalyst followed by oxidative addition, ligand exchange, and reductive elimination. The scope of this procedure was investigated later on in details by the Sutherland group who demonstrated its generality (Cant et al., 2012).

**Figure 4:**
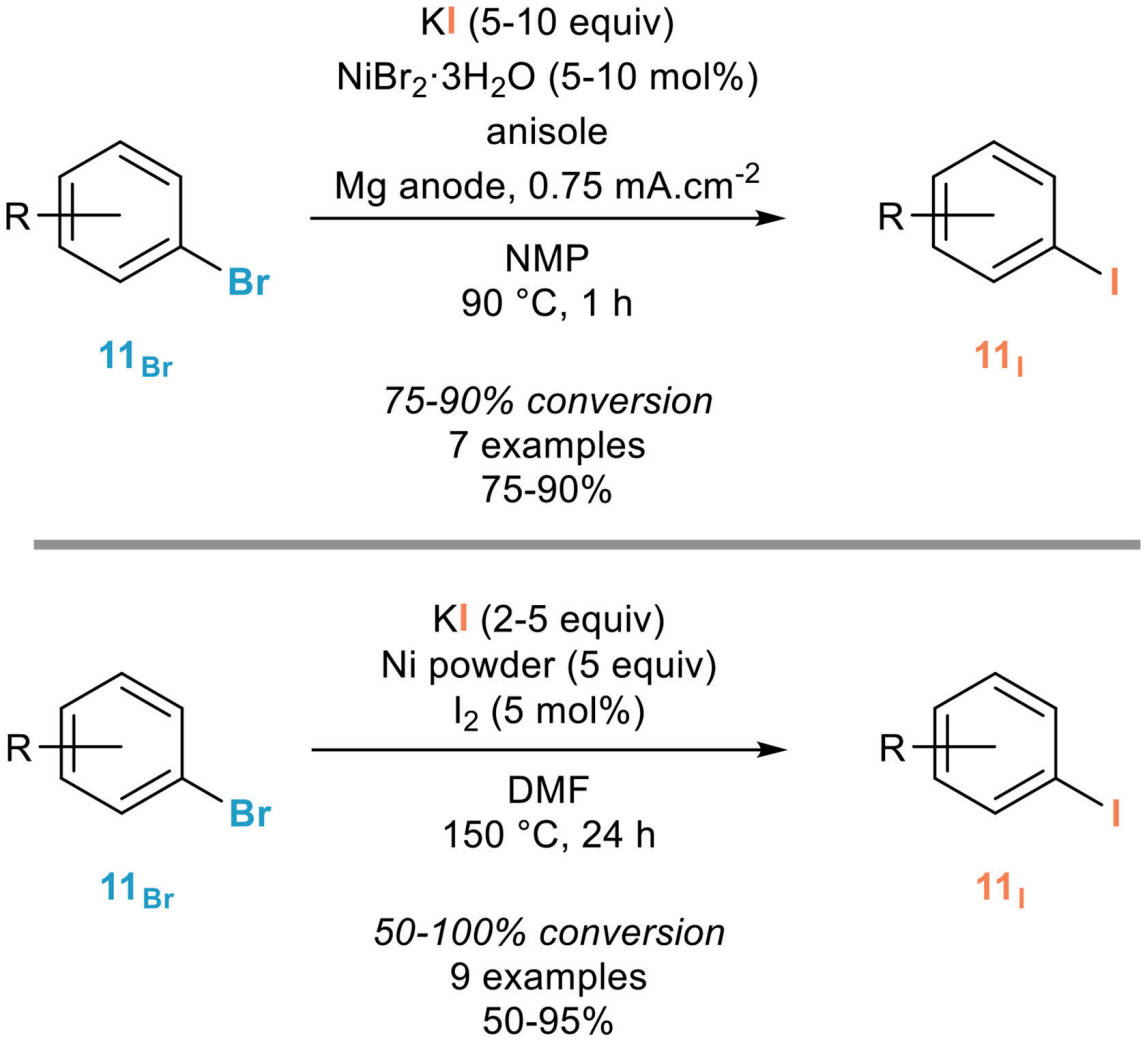
Use of metallic or electro-generated nickel(0) in the aromatic Finkelstein reaction.

Assuming that zinc acts as a reducing agent favoring the *in situ* formation of nickel(0) complexes, the Perichon group demonstrated eight years later that the electrochemical reduction of nickel bromide in the presence of potassium iodide leads to such nickel(0) complexes which were shown to efficiently catalyze the halogen exchange of a series of non-activated aryl bromides **11**_Br_ to the corresponding iodides **11**_I_ in NMP at 90°C, especially in the presence of additional anisole whose beneficial use was not rationalized (Figure 4, top; Meyer et al., 1986). However, a brief study of the scope and limitations of this process revealed that full conversions were not obtained in most cases, which represents a strong limitation due to the close physicochemical properties of the starting materials and products. Increasing the reaction time resulted in much lower yields due to electroreducing dimerization of the aryl iodide **11**_I_ formed in the presence of the nickel(0) catalyst.

A related process was reported the next year by the Cheng group who showed that nickel(0) powder, used in large excess, could efficiently mediate the aromatic Finkelstein reaction of a range of aryl bromides **11**_Br_ (Figure 4, bottom; Yang et al., 1987). Pretreatment of the nickel powder with small amounts of molecular iodine was shown to have a great effect on the rate and efficiency of the halogen exchange and although five equivalents of this powder was used, the authors demonstrated that it could be successfully reused more than ten times without significant loss of activity. However, as in the previous case, the reaction was shown to be highly substrate-dependent and did not reach completion in many cases. The use of chlorinated arenes turned out to be less efficient and the authors proposed a mechanism involving first chemisorption of the aryl bromide **11**_Br_ to the nickel surface followed by nucleophilic attack of iodide facilitated by the coordination of the arene to the surface, expulsion of the bromide and desorption of the resulting aryl iodide **11**_I_.

As a note, the more challenging aromatic Finkelstein reaction from aryl chlorides to the corresponding bromides was found to be effective by reacting the former with two equivalents of nickel(II) bromide in DMF at 170°C under microwave irradiation, although with moderate yields ranging from 41 to 45% (Arvela and Leadbeater, 2003).

Considering the mechanism of these nickel-mediated or catalyzed aromatic Finkelstein reactions, there is no clear cut evidence in favor of one of the mechanisms overviewed in Figure 2 and different mechanisms might actually be at play depending on the catalysts used (and their oxidation states) and reaction conditions. Indeed, oxidative addition/reductive eliminations (Takagi et al., 1980; Tsou and Kochi, 1980; Cant et al., 2013), single electron transfers (Bozell and Vogt, 1988) and σ-bond metathesis (Tsou and Kochi, 1980) have been proposed, although with no experimental support in most cases.

As evidenced with these representative examples, while nickel complexes could be successfully used to demonstrate the feasibility of an aromatic Finkelstein reaction, the main problems associated with these complexes are the requirement of rather harsh conditions and highly polar solvents and, which is the main limitation, non-total conversions. These drawbacks prompted various research groups to consider other metal complexes as catalysts: among all metals that have been evaluated, palladium was shown to be somehow efficient but the reaction was only used for the *in situ* generation of aryl iodides from the corresponding chlorides (Thathagar and Rothenberg, 2006) and the use of gold complexes requires either specific macrocyclic substrates or the presence of a strongly chelating group *ortho* to the halogen atom (Serra et al., 2015). Copper proved to be one of the most efficient metals: copper-catalyzed aromatic Finkelstein reactions will be overviewed in the next paragraphs.

#### Catalyzed/mediated by copper complexes

**Figure 5:**
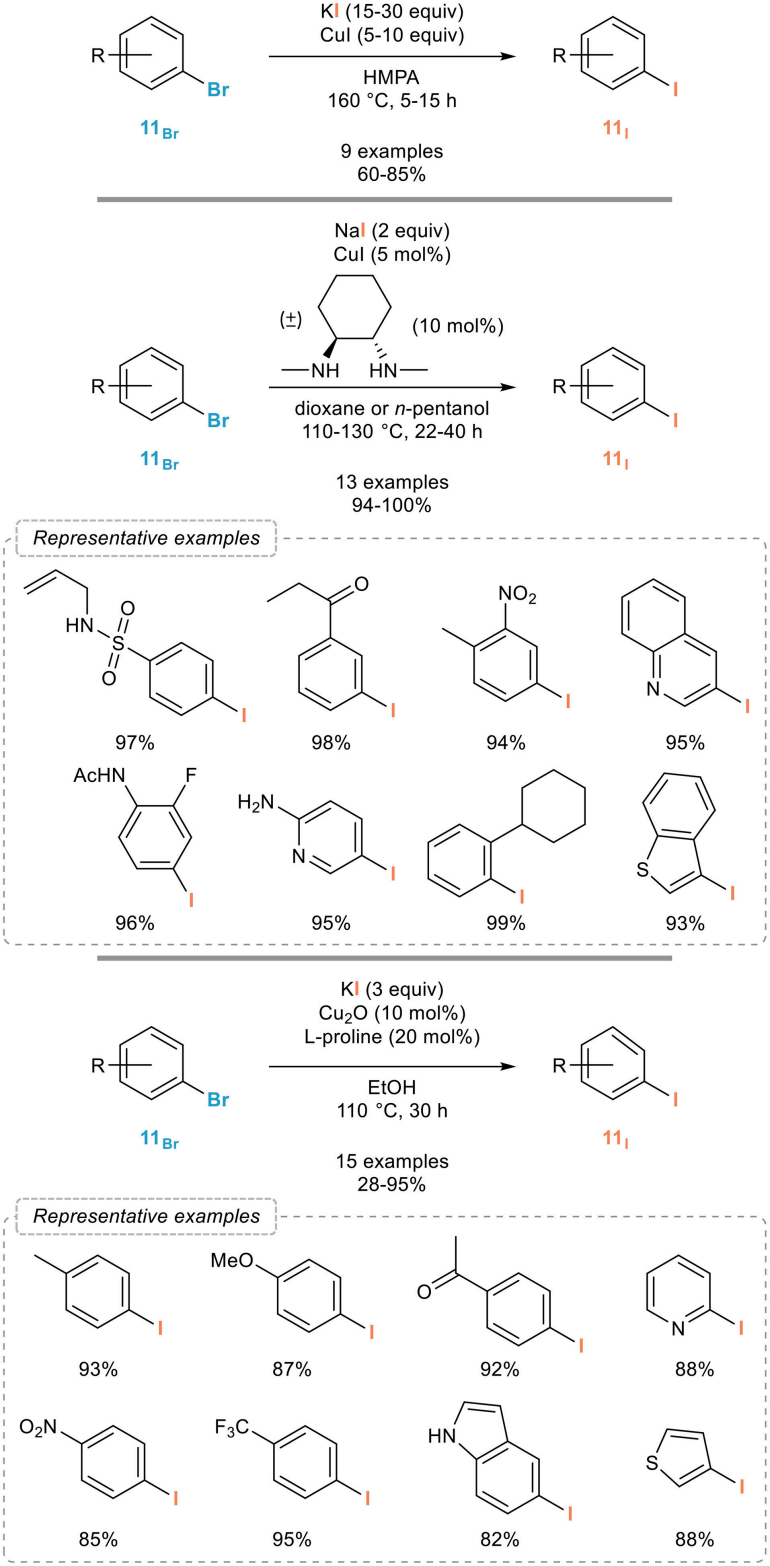
Copper-mediated/catalyzed aromatic Finkelstein reaction.

Copper complexes were indeed shown to be remarkable promoters for the aromatic Finkelstein reaction (Casitas and Ribas, 2013b). The very first example was reported by the Suzuki group in 1985 and relied on Ullmann-Goldberg type conditions (Figure 5, top; Suzuki et al., 1985, 1986). Upon reaction with an excess of copper(I) iodide and potassium iodide in HMPA at 160°C, a series of aryl iodides **11**_I_ could be readily obtained from the corresponding bromides **11**_Br_ in fair to good yields and regardless to the substitution pattern and electronic properties of the starting materials. Importantly, full conversions were obtained in all cases. As a note, it was shown in 2012 that HMPA could be replaced by the less toxic DMI, (Yamashita et al., 2011) although the significance of this modified procedure might be low when compared to the development of ligand-assisted, copper-catalyzed versions of this aromatic Finkelstein reaction.

If these pioneering results by the Suzuki group demonstrated the possibility of using copper complexes to promote the aromatic Finkelstein reactions, this process indeed however suffers from the use of excess copper salts and drastic reaction conditions. A much more general and efficient process was reported more than fifteen years later by the Buchwald group. In continuation of their studies on the use of diamine-copper complexes as catalysts for cross-coupling reactions, (Surry and Buchwald, 2010) they reported in 2002 one of the most broadly applicable process for the aromatic Finkelstein reaction (Figure 5, middle; Klapars and Buchwald, 2002). Upon reaction with sodium iodide and catalytic amounts of copper(I) iodide and (±)-*trans*-*N*,*N*′-dimethyl-1,2-cyclohexanediamine in dioxane at 110°C, a variety of aryl bromides **11**_Br_ could be smoothly converted to their iodinated analogs **11**_I_ with excellent yields. The scope of the reaction was shown to be remarkably broad and this process clearly is one of the most efficient to date, as exemplified with representative synthetic application of this process that will be overviewed in section Synthetic Applications of Halogen Exchanges in Aryl Halides and its use in natural product synthesis (Fürstner and Kennedy, 2006; Carr et al., 2010). It was later on extended to other (hetero)aryl bromides (Lützen et al., 2014) and to continuous flow chemistry using a sodium iodide packed-bed reactor (Chen et al., 2014) and recent studies demonstrated that the reaction time could be shortened using microwave irradiation (Cannon et al., 2011) and that other diamines and some triamines (Jin and Davies, 2017) could also be used as ligands. Aryl chlorides are in general poor substrates in this reaction. An alternative catalytic system that might be useful to consider should the Buchwald's procedure be troublesome with some substrates is the one reported by the Bao group in 2016 (Figure 5, bottom). They indeed reported that a combination of copper(I) oxide and L-proline in ethanol could also efficiently catalyze the aromatic Finkelstein reaction from all kind of aryl bromides **11**_Br_ and with high efficiency (Feng et al., 2016).

**Figure 6:**
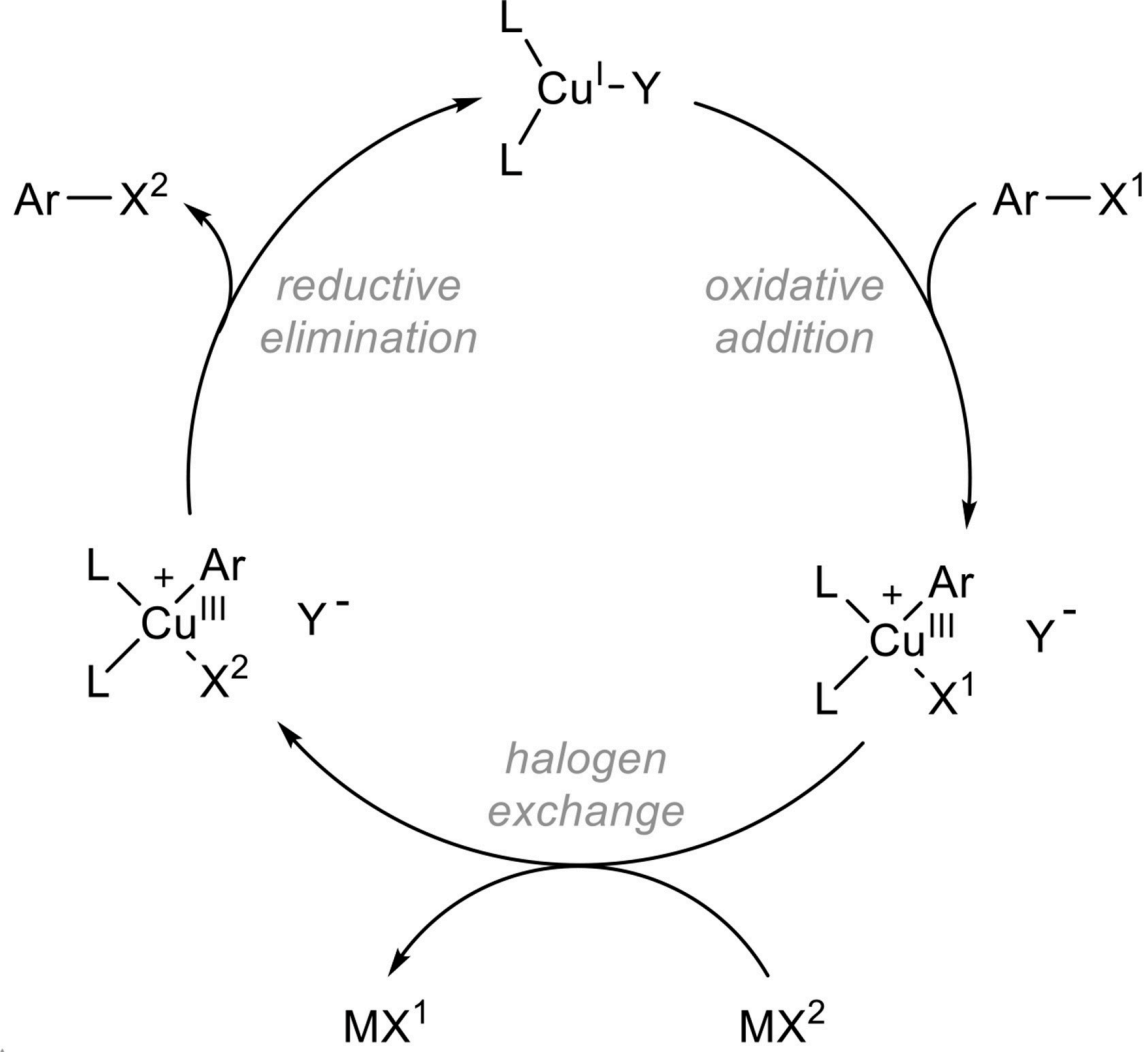
Proposed mechanism for the copper-catalyzed aromatic Finkelstein reaction.

From a mechanistic point of view, these reactions might proceed by oxidative addition at the copper(I) catalyst yielding a transient arylcopper(III) complex that would then undergo a halide exchange followed by reductive elimination as shown in Figure 6, the driving force being the precipitation of sodium/potassium bromide. Elegant mechanistic studies by the Ribas group using macrocyclic arylcopper(III) model systems support this mechanism (Casitas et al., 2011) although alternative pathways might be operative in some cases (Casitas and Ribas, 2013a).

Among all metals evaluated to date to promote the aromatic Finkelstein reaction, copper is by far the most efficient one. Capitalizing on the fact that light-induced radical processes could be also utilized for exchanging halogen atoms in aryl halides, a recent report in this area disclosed a rather efficient process for the photo-induced aromatic Finkelstein reaction: it will be briefly overviewed in the next section.

#### Photo-induced

**Figure 7:**
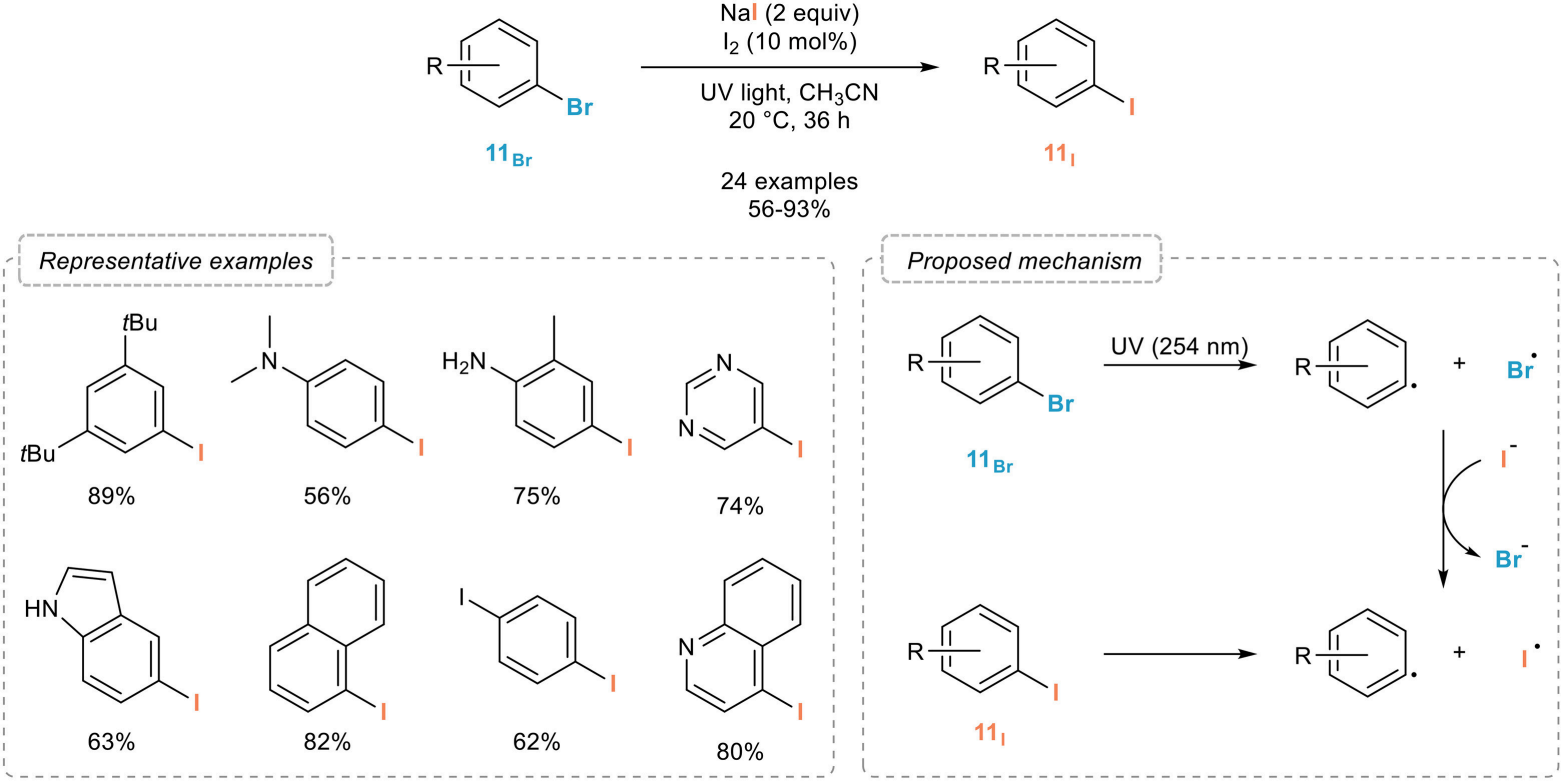
Photo-induced aromatic Finkelstein reaction.

Indeed, in order to develop a metal-free iodination of aryl bromides **11**_Br_ and based on their absorption of UV light, Li and coworkers reported in 2015 an efficient process for the photo-induced aromatic Finkelstein reaction (Figure 7; Li et al., 2015). Upon simple irradiation of the starting aryl bromides **11**_Br_ with UV light in the presence of sodium iodide and a catalytic amount of molecular iodine in acetonitrile at room temperature, a clean and broadly applicable halogen exchange occurs providing the corresponding aryl iodides **11**_I_ in good to excellent yields. Interestingly, the reaction could also be extended to the more challenging iodination of aryl chlorides, although with reduced efficiency. As for the mechanism of this transformation, the authors suggested a light-induced homolytic fragmentation of the carbon-bromide bond in the starting aryl bromide **11**_Br_ yielding intermediate aryl and bromine radicals. The latter would then further oxidize iodine anions to form iodine radicals whose recombination with the aryl radical would produce the corresponding aryl iodides **11**_I_.

As evidenced with the results overviewed in this section, the development of catalytic systems to facilitate the aromatic Finkelstein reaction has attracted the attention of various research groups, which resulted in the design of remarkably efficient processes enabling the transformation of aryl bromides to less available, more expensive, and usually more reactive aryl iodides. The extension of these processes to aryl chlorides, which would be an especially useful addition due to their greater availability, is still challenging and clearly deserves to be further explored since there is quite some room for improvement.

The reverse reaction, i.e., the formal replacement of a halogen atom by a lighter one, has also been extensively studied: the next section of this review article will be devoted to an overview of the most efficient systems to promote the aromatic retro-Finkelstein reaction.

### Aromatic retro-finkelstein reactions

While the synthetic usefulness of the aromatic Finkelstein reaction, which enables the preparation of aryl iodides from their more available brominated and, to a lesser extent, chlorinated counterparts, is quite obvious, the reverse reaction is also attractive, notably for late-stage modifications and for the introduction of fluorine (which will be overviewed in section Metal-Mediated Fluorination of Aryl Halides). In this case, the driving force of the reaction is the formation of a stronger carbon-halogen bond.

Since the mechanisms involved are actually the same as those involved in all reactions described in the previous section (they will therefore not be overviewed again in this section for the sake of conciseness), it is quite logical that similar metal complexes can also be used to promote the aromatic retro-Finkelstein reaction. Silver complexes have also been reported for this transformation, but only with specific macrocyclic substrates (Font et al., 2014).

#### Catalyzed/mediated by nickel complexes

**Figure 8:**
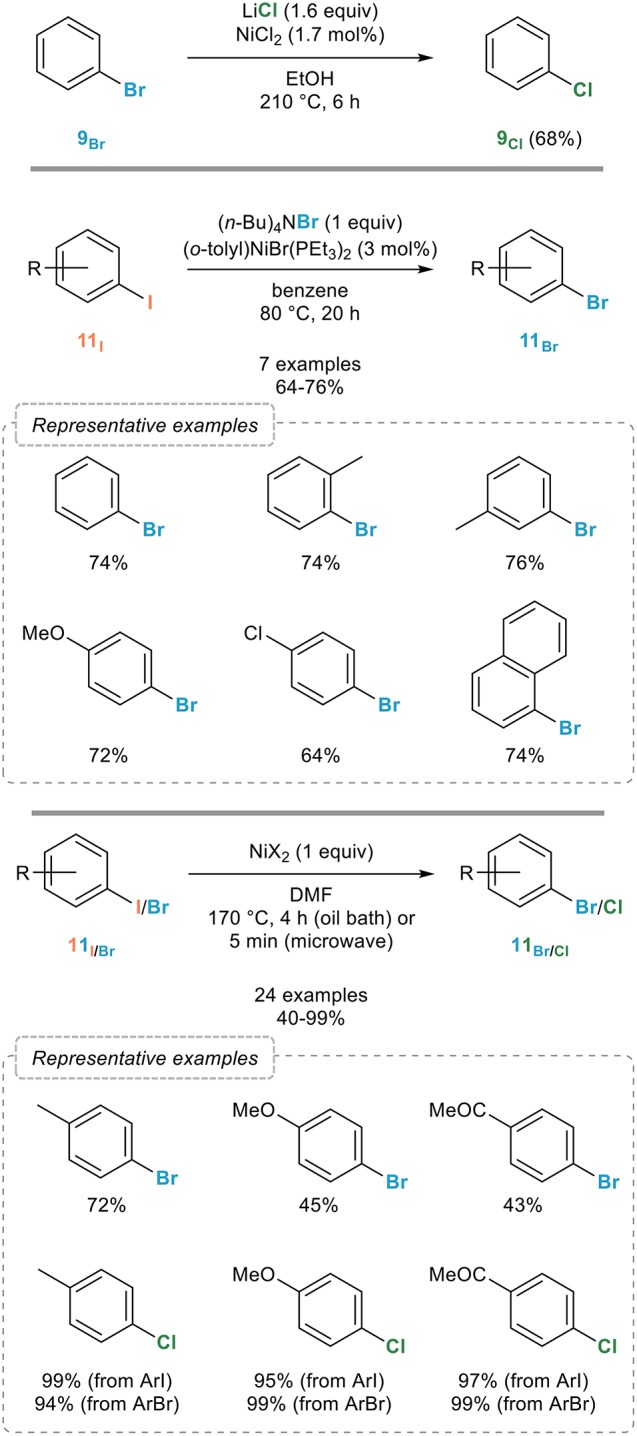
Nickel-catalyzed aromatic retro-Finkelstein reaction.

Nickel complexes were also the first ones to be reported to promote this reaction which was actually disclosed before the reverse process. Indeed, Cramer and Coulson reported in the mid-1970s that bromobenzene **9**_Br_ could be transformed to chlorobenzene **9**_Cl_ upon reaction with a catalytic amount of nickel(II) chloride in the presence of lithium chloride in DMF at 210°C for 6 h (Figure 8, top; Cramer and Coulson, 1975).

A more general system operating under milder conditions was reported five years later by the Kochi group who screened a series of nickel complexes to promote this reaction (Tsou and Kochi, 1980). While various nickel complexes with different oxidation states ranging from 0 to +II were found to be efficient (pre)catalysts, other were found to be totally ineffective, which was attributed to the ease with which they are converted under the reaction conditions to nickel(I) species, assigned as catalytic ones. The most efficient catalytic system was found to rely on the use of (*o*-tolyl)NiBr(PEt_3_)_2_ in combination with tetrabutylammonium bromide as the brominating agent which enabled a smooth bromination of a range of aryl iodides **11**_I_ (Figure 8, middle). Mechanistic studies revealed an inhibition of the halogen exchange by quinones and nitroarenes, which is in favor of a radical chain process, aryl halides having the intrinsic propensity to undergo radical *ipso* substitution reactions. Based on this mechanism, the Burrows group reported in 1991 an alternative system for the chlorination of aryl iodides and bromides based on the generation of Cl^•^ by bleach and catalytic amounts of nickel(II) tetraphenylporphyrinate in the presence of benzyltributylammonium bromide as the phase transfer catalyst (O'Connor and Burrows, 1991).

An improved, although not catalytic, procedure was reported some twenty years later by the Leadbeater group who demonstrated that simply heating an aryl iodide **11**_I_ or bromide **11**_Br_ with two equivalents of nickel(II) bromide or chloride in DMF at 170°C for 4 h smoothly provided the corresponding aryl bromides **11**_Br_ or chlorides **11**_Cl_ in good to excellent yields (Figure 8, bottom; Arvela and Leadbeater, 2003). The halogen exchange can be performed equally well using conventional (4 h) or microwave (5 min) heating and is not sensitive to oxygen.

#### Mediated by palladium complexes

**Figure 9:**
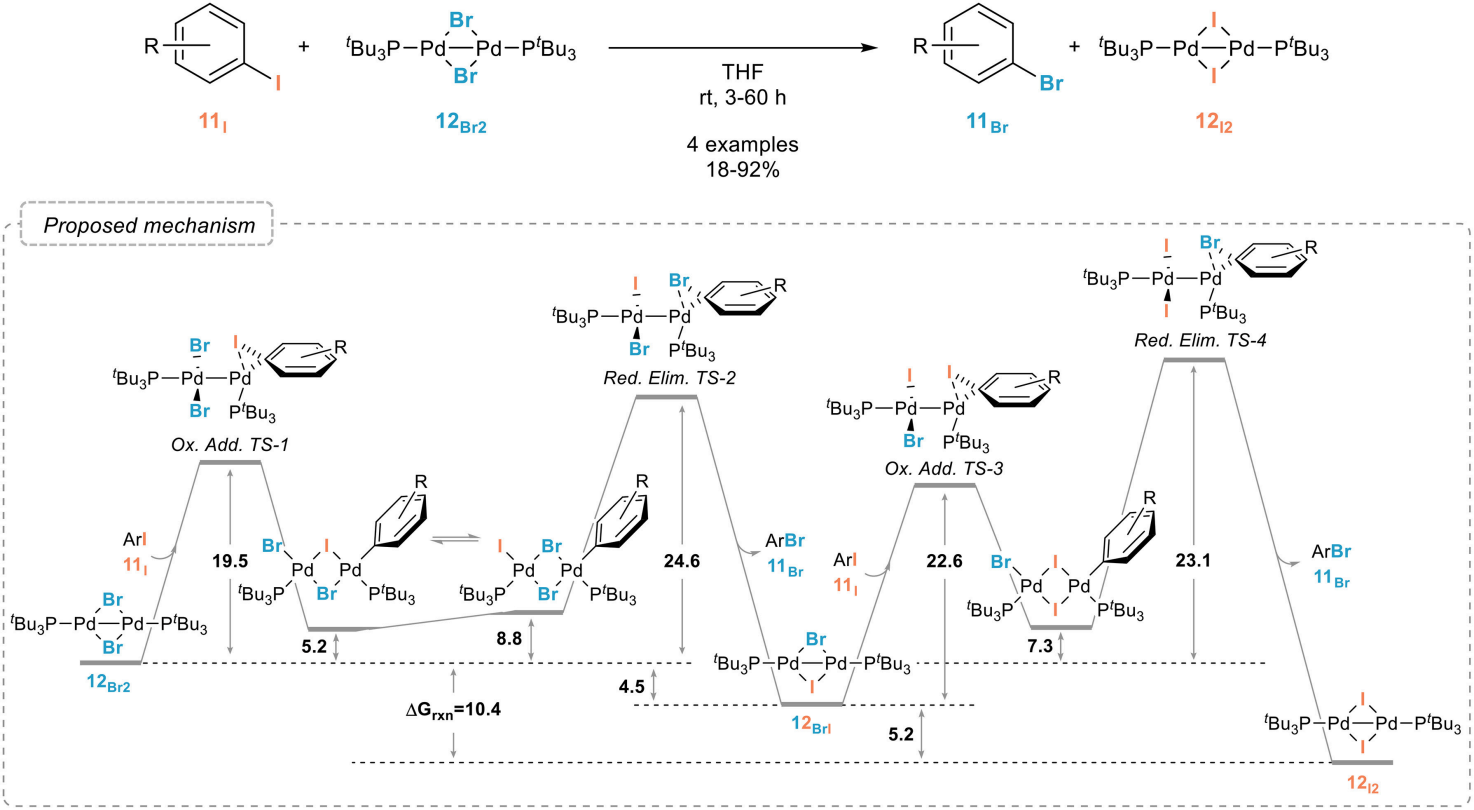
Palladium(I) dimer-mediated aromatic retro-Finkelstein reaction and mechanism associated (values are in kcal mol^−1^).

In sharp contrast to nickel, palladium complexes have been only scarcely studied as catalysts for the aromatic retro-Finkelstein reaction. While they have been shown to be especially efficient for the fluorination of aryl halides, their use for simpler halogen exchange reactions seem to have been, for some obscure reasons, quite neglected. A recent report from the Schoenebeck group however demonstrated their potential, even if these studies were mostly focused on the mechanism of the halogen exchange rather than on the development of a synthetically useful procedure. A dinuclear palladium(I) complex with bridging bromides **12**_Br_ was indeed shown to readily react with aryl iodides **11**_I_, yielding the corresponding iodinated palladium(I) dimer **12**_I_ and aryl bromide **11**_Br_ (Figure 9). (Bonney et al., 2013; Kalvet et al., 2014). Interestingly, and which might account for the limited number of studies on palladium-catalyzed halogen exchange processes, the halogen exchange could not be achieved through analogous palladium(0) conditions, which could be attributed, after extensive kinetic and computational studies, to the unique reactivity of the palladium(I) dimer. The free energy pathway associated to this reaction and to the oxidative additions/reductive eliminations involved is shown in Figure 9.

As with the Finkelstein reaction, its reverse reaction can be most efficiently catalyzed by copper complexes which are to date still among the most efficient catalysts. The most significant advances in the copper-catalyzed aromatic retro-Finkelstein reaction will be overviewed in the following paragraphs.

#### Catalyzed by copper complexes

The first example of a copper-mediated aromatic retro-Finkelstein reaction actually dates back to 1958 when Fortenbaugh reported that “the replacement of bromine by chlorine in aromatic compounds” could be effected by using an excess cuprous chloride (Hardy and Fortenbaugh, 1958). While this demonstrated the feasibility of such a process, the substrate used for this study, 2-acetamido-3-bromoanthraquinone, is a really specific one and in addition benefits from a strong *ortho* effect. Systematic studies by Bacon and Hill relying on the use of excess cuprous chloride in highly polar solvents next generalized this reaction (Bacon and Hill, 1964a,b), and further studies by the Ribas group with macrocyclic substrates next demonstrated the possibility of a retro-Finkelstein reaction catalytic in copper, still with specific substrates however (Casitas et al., 2011).

**Figure 10:**
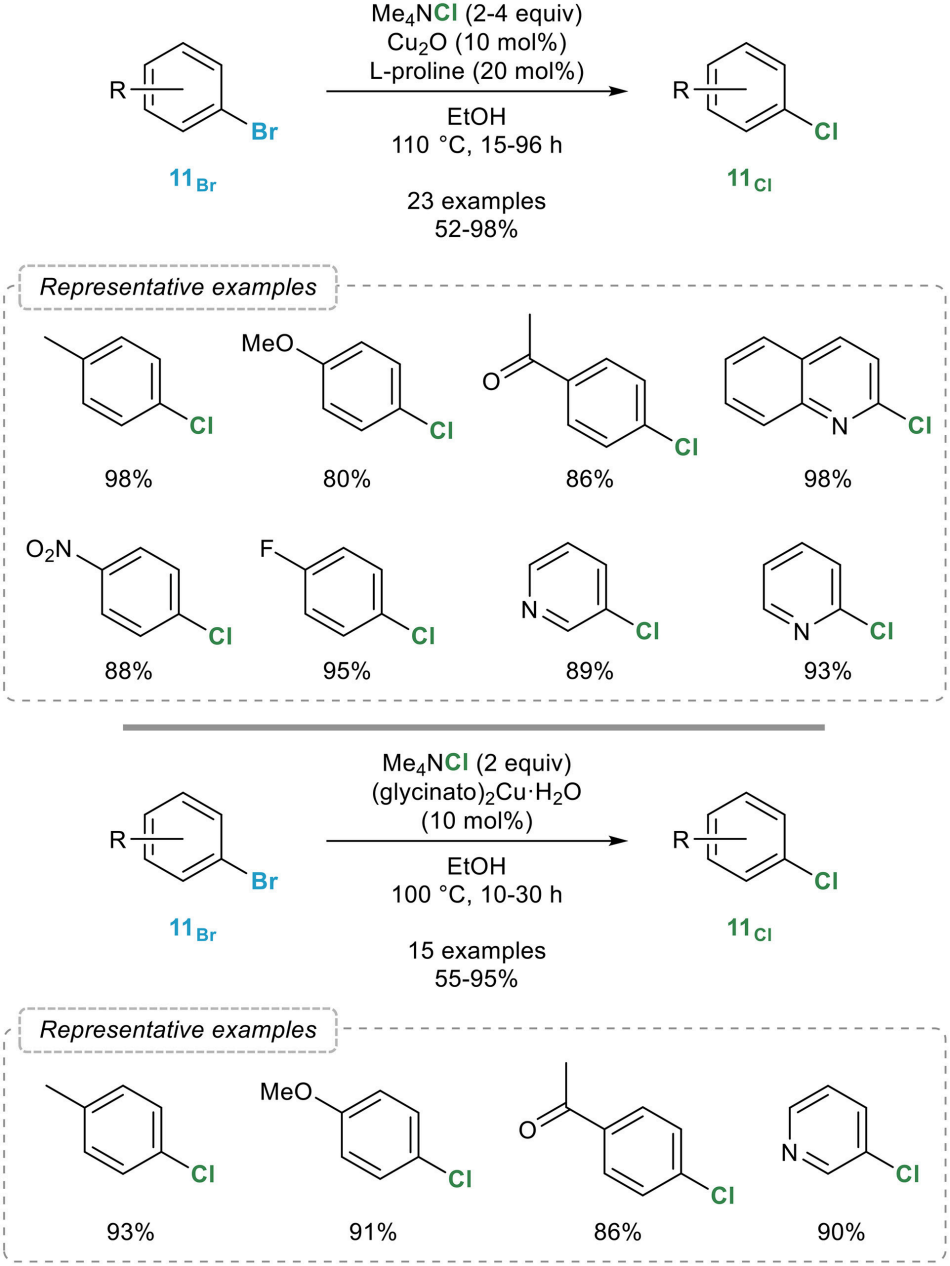
Copper-mediated/catalyzed aromatic retro-Finkelstein reaction.

The first general procedure for the catalytic aromatic retro-Finkelstein reaction was reported in 2012 by the Bao group (Feng et al., 2012) who showed that a combination of catalytic amounts of copper(I) oxide and L-proline in ethanol smoothly catalyzed the bromine to chlorine exchange with tetramethylammonium chloride in a series of aryl bromides **11**_Br_ with excellent yields (Figure 10, top). The electronic properties of the starting bromide were found to have little influence on the outcome of the reaction and among all different sources of chlorine evaluated, tetramethylammonium chloride, which is soluble in ethanol—a prerequisite for the reaction to occur—was found to be highly superior. Close conditions were reported by the Jain group two years later (Verma et al., 2014) who simply replaced the combination of copper(I) oxide and L-proline reported by Bao by a preformed bis(glycinato)copper(II) complex, most certainly reduced *in situ* to the corresponding copper(I) catalyst (Figure 10, bottom). While really close to Bao's catalytic system, the main advantage of this preformed precatalyst probably lies in its reusability since it could be easily recovered by filtration after completion of the reaction and reused without significant loss of activity. Moreover, ICP-AES analysis of crude reaction mixtures indicated no leaching of the catalyst.

As with the Finkelstein reaction, the propensity of aryl halides to undergo radical *ipso* substitution reactions led to the development of photo-induced processes. The single one reported to date for the retro-Finkelstein reaction will be overviewed in the following section.

#### Photo-induced

**Figure 11:**
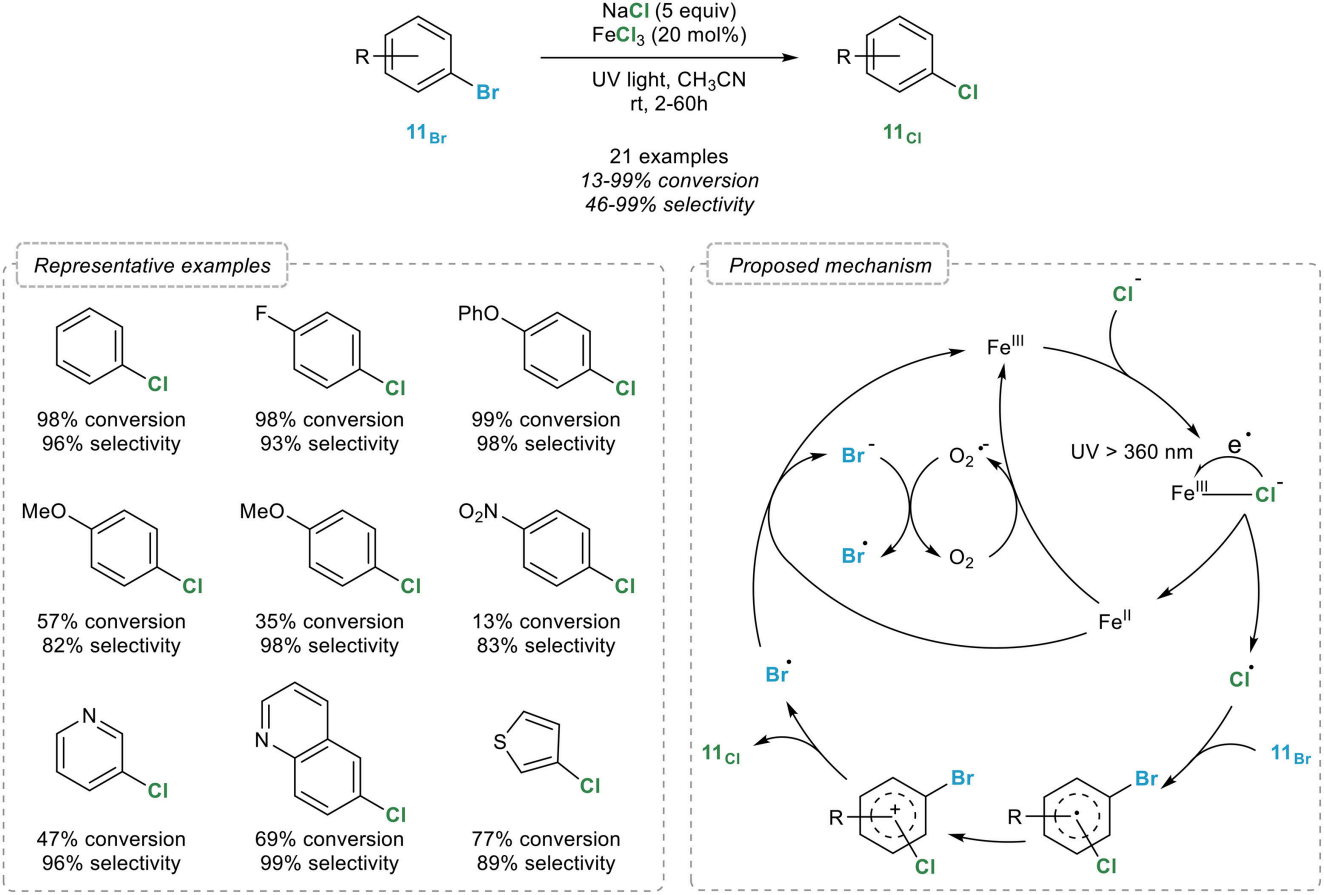
Photo-induced aromatic retro-Finkelstein reaction.

The Chen group indeed reported in 2014 an efficient iron-catalyzed, photo-induced chlorination of aryl bromides **11**_Br_ with an attractive source of chloride: sodium chloride (Wang et al., 2014). Upon reaction with an excess of sodium chloride and 20 mol% of iron(III) chloride in acetonitrile under irradiation with UV light, a broad range of (hetero)aryl bromides **11**_Br_ were readily converted to their chlorinated analogs (Figure 11). While the selectivity is high in most cases, it does however depend on the nature of the substrate and can decrease with increased conversions, which somehow reduces the synthetic potential of this process. The authors noted that the reaction could be performed under argon or oxygen, the halogen exchange being actually faster in this case. In order to rationalize the influence of oxygen, two mechanisms have been proposed by the authors. In both cases, irradiation of an iron(III) chlorine complex would initiate the cleavage of the Fe-Cl bond, generating an iron(II) intermediate and a Cl^•^ radical which would next perform the bromide to chlorine exchange *via* a radical *ipso* substitution reaction that would in turn release a Br^•^ radical. The reaction with this free radical and the iron(II) intermediate would lead to the regeneration of the iron(III) catalyst with the concomitant formation of a bromine ion. In the case of aerobic reaction, the acceleration noted could be attributed to a rapid oxidation of the iron(II) intermediate with molecular oxygen leading to the regeneration of the iron(III) catalyst and a superoxide ${\text{O}}_{2}^{•}$ radical which would finally oxidize the Br^•^ radical into bromine.

As evidenced with all examples commented above, a set of efficient procedures have been recently developed enabling the halogen exchange in a broad range of substrates with high efficiency. Aryl iodides can indeed be efficiently converted to their lighter brominated and chlorinated homologs and aryl bromides to the corresponding iodinated and chlorinated arenes. The transformation of aryl chlorides to their heavier homologs is still challenging, even if some procedures have been recently reported to perform such reactions: efforts are however clearly needed for the development of robust and broadly applicable processes. The next section of this review article will be devoted to the fluorination of aryl halides, a major transformation for the synthesis of fluorinated arenes that has attracted the attention of various research groups worldwide and for which remarkably efficient procedures have been recently reported.

### Metal-mediated fluorination of aryl halides

Aryl fluorides are indeed key building blocks relevant to many areas of science, especially in medicinal chemistry and agrochemistry. This is readily evidenced by the ever-growing number of drugs containing such motifs—the most striking examples certainly being the top-selling drugs Lipitor (atorvastatin), Lexapro (escitalopram), Crestor (rosuvastatin), or Zetia (ezetimibe)—which implies the development of efficient methods for the introduction of a fluorine atom into an aromatic core (Maienfisch and Hall, 2004; Thayer, 2006; Purser et al., 2008; O'Hagan, 2010; Gillis et al., 2015). Moreover, the potential extension of such processes to the introduction of ^18^F is especially relevant in medical imaging, radioactive ^18^F-labeled organic compounds being widely used as contrast agents for positron emission tomography (PET). Among all methods reported to date to perform such a transformation, the selective replacement of a halogen atom by a fluorine is one of the most attractive ones, although quite challenging. Two main metals have been reported to catalyze the fluorination of aryl halides, palladium and copper: their use for the fluorination of aryl halides will be overviewed in the following paragraphs.

#### Catalyzed by palladium complexes

The use of transition metals for the displacement of a halide in readily available and/or easily accessible chloro-, bromo-, and iodo- arenes with fluoride—which is thermodynamically favored but kinetically disfavoured in the absence of a catalyst due to the too high energy of the Meisenheimer complexes that would be involved starting from non-activated haloarenes—is indeed one of the most attractive methods for the synthesis of aryl fluorides. While palladium catalysis has been remarkably successful for the formation of C_Ar_-N and C_Ar_-O bonds, its use for the fluorination of aryl halides turned out to be challenging. It does indeed involve an oxidative addition followed by ligand exchange and reductive elimination: while the first elementary step cannot fail, the feasibility of the two other ones could be questioned due to the lack of data available on arylpalladium(II) fluorides. Remarkable studies from Grushin on the synthesis and reactivity of such complexes revealed the high energy barrier for the reductive elimination and problems associated with the formation of fluoride-bridged dimers and formation of P-F containing side products (Grushin, 2010).

**Figure 12:**
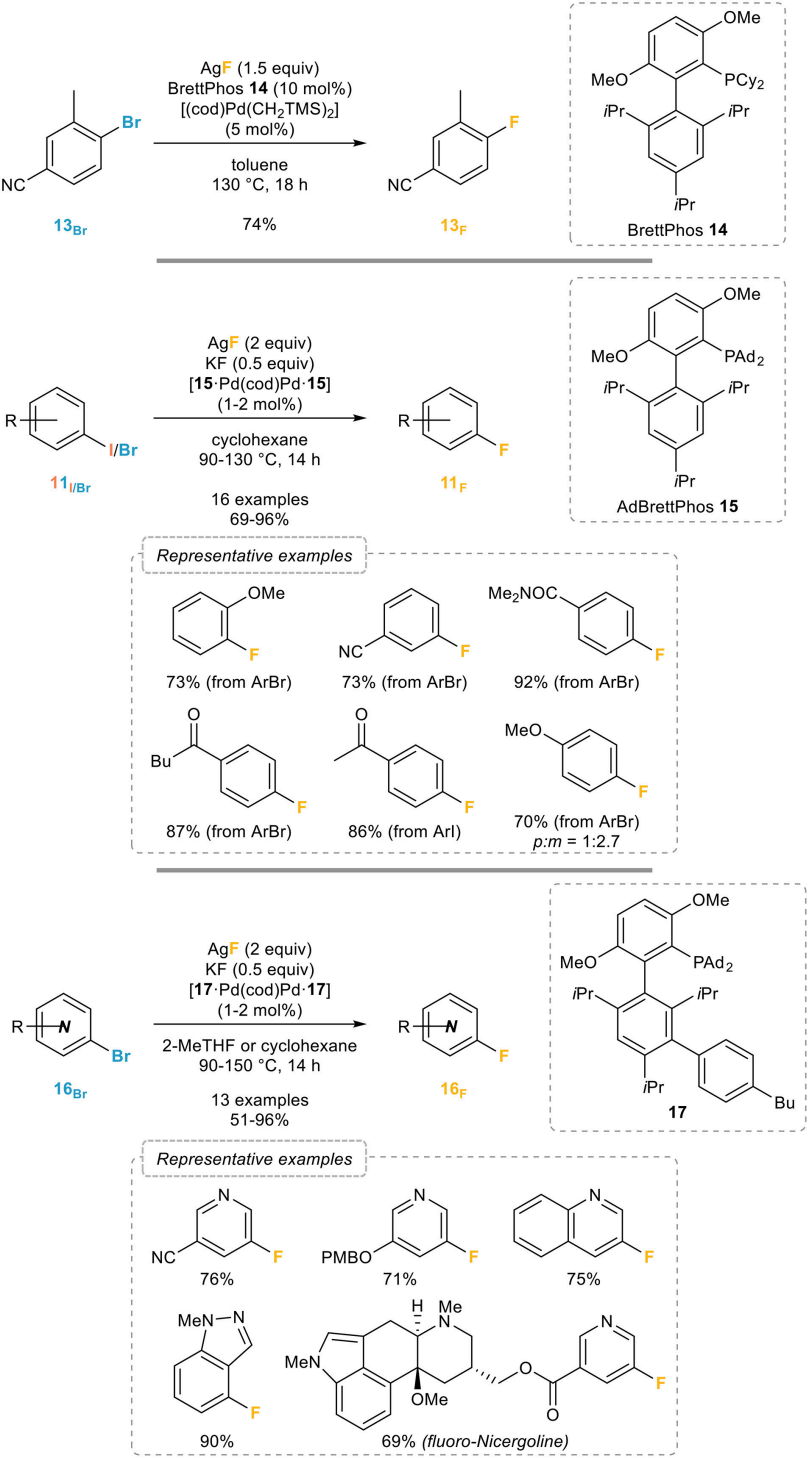
Palladium-catalyzed fluorination of aryl halides.

A major step forward in this area was reported by the Buchwald group in 2009 (Watson et al., 2009) who demonstrated that halide exchange and reductive elimination were all possible by using their BrettPhos ligand (Figure 12, top). 1-Bromo-4-cyano-toluene **13**_Br_ could be successfully transformed to its fluorinated analog **13**_F_ in 74% yield using silver fluoride as the fluorinating agent and a combination of [(cod)Pd(CH_2_TMS)_2_] and Brettphos **14** in toluene at 130°C. However, only highly activated (i.e., *ortho*-substituted and electron-deficient) aryl bromides could be successfully fluorinated. Based on the observation that *in situ* ligand arylation occurred during the related catalytic fluorination of aryl triflates and that the catalyst is effective only when it is supported by the arylated ligand, which could be facilitated by the presence of an additional base, the Buchwald group (Lee et al., 2014) reported a general system for the catalytic fluorination of aryl bromides in 2014 (Figure 12, middle). Upon reaction with catalytic amounts of dimeric precatalyst based on AdBrettPhos **15**, 2 equivalents of silver fluoride, substoichiometric amounts of potassium fluoride in cyclohexane at temperatures ranging from 90 to 130°C, a broad range of aryl iodides **11**_I_ and bromides **11**_Br_ could be readily transformed into the corresponding aryl fluorides without significant reduction, a side reaction commonly observed in such transformations. In the case of electron-rich aryl halides lacking *ortho* substituents such as 4-bromo-anisole, mixtures of regioisomers are however obtained, which is certainly the main limitation of this process. Attempts at extending these conditions to the fluorination of heteroaryl bromides **16**_Br_ failed to provide the fluorinated heteroarenes **16**_F_ in satisfactory yields, a problem that could be circumvented by the use of a precatalyst with a new ligand **17** that had been modified *ex situ*. This new precatalyst turned out to be remarkably efficient, when used in combination with both silver and potassium fluorides, for the fluorination of various heteroaryl bromides **16**_Br_, therefore providing a most efficient route to heteroaryl fluorides **16**_F_ (Figure 12, bottom). Further catalyst design eventually led to a fluorinated analog of this ligand, “AlPhos,” that enables the room-temperature and regioselective palladium-catalyzed fluorination of aryl bromides (Sather et al., 2015).

While palladium catalysis has clearly provided the most efficient methods reported to date for the catalytic fluorination of aryl halides, a lot of other metals have also been investigated. While most of them gave unsatisfactory results, copper complexes, often used in stoichiometric amounts, recently provided efficient alternatives to palladium catalysis. The most significant results in this area are overviewed in the next section.

#### Catalyzed/mediated by copper complexes

**Figure 13:**
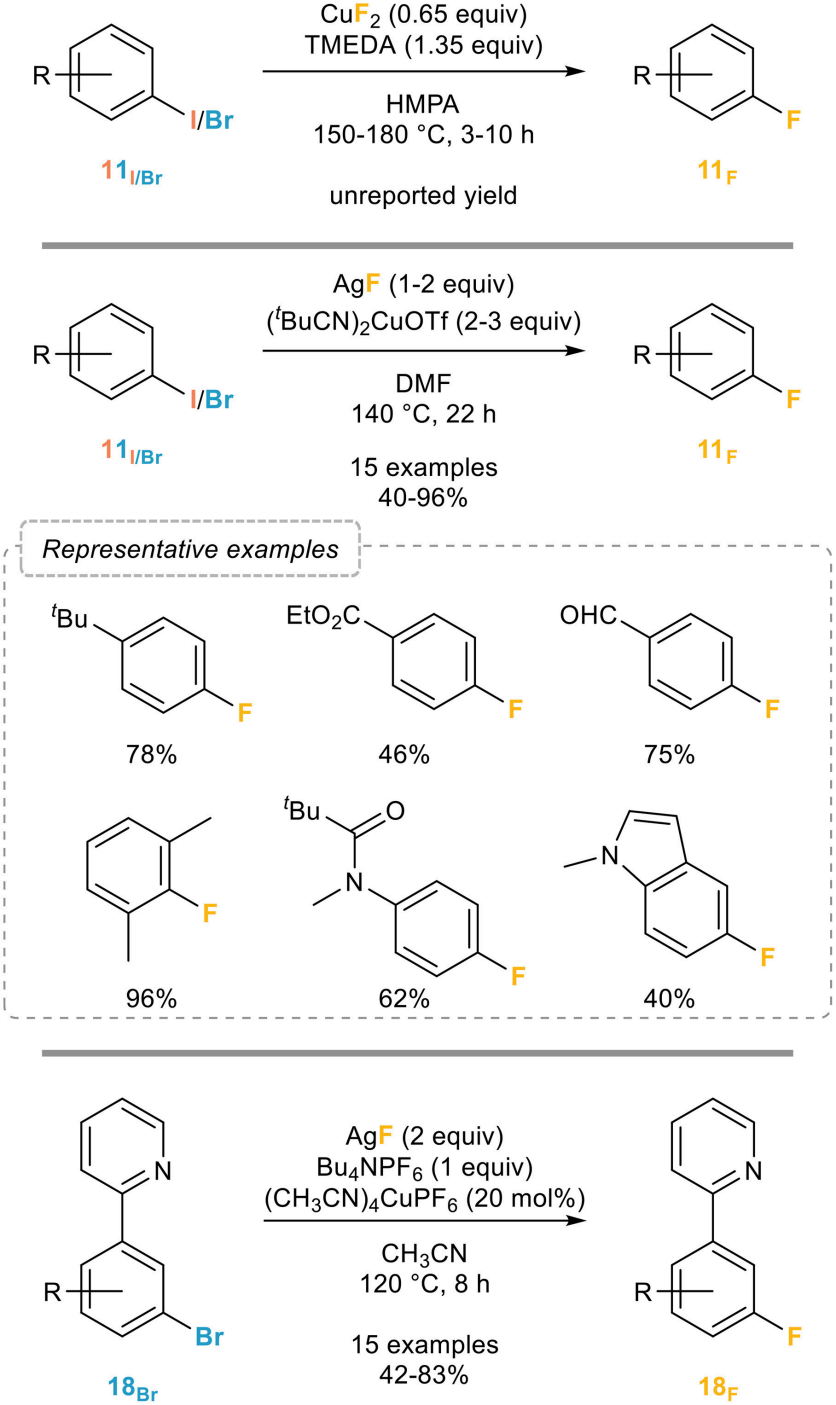
Copper-mediated/catalyzed fluorination of aryl halides.

The Grushin group actually also pioneered this area and patented in 2007 (Grushin, 2007) a process for the fluorination of a wide range of unactivated aryl halides by using a slight excess of copper(II) fluoride and TMEDA in HMPA at high temperatures (Figure 13, top). While this method is quite remarkable and efficient, clear drawbacks are the use of stoichiometric copper(II) fluoride and the rather harsh conditions.

A major breakthrough toward the development of a more robust and general process was reported by the Hartwig group in 2012 (Fier and Hartwig, 2012). Inspired by the work of the Ribas (Casitas et al., 2011) group who demonstrated the possibility of a reductive elimination from an arylcopper(III) fluoride and based on the assumption that this reductive elimination would be favored by a non-coordinating counterion and weakly donating ligands, a series of cationic copper complexes ligated by nitriles to promote the fluorination was evaluated, which eventually led to the development of efficient conditions based on the use of 3 equivalents of (^*t*^BuCN)_2_CuOTf and 2 equivalents of silver fluoride in DMF at 140°C (Figure 13, middle).

While clearly efficient and quite general, the main limitation of the Hartwig's procedure is the requirement of an excess of the copper complex and still quite harsh reaction conditions. While a catalytic procedure would clearly be more appealing, all efforts toward the development of such a process have failed, except when specific substrates were used. Indeed, the only fluorination of brominated arenes catalytic in copper reported to date was published in 2014 by the Liu group who managed to perform a catalytic fluorination of 2-pyridyl-bromobenzene derivatives based on the use of catalytic amounts of Kubas complex together with silver fluoride and tetrabutylammonium hexafluorophosphate as an additive (Figure 13, bottom). While efficient, broadly applicable and quite insensitive to the substitution pattern and electronic properties of the starting materials, the presence of the chelating pyridine is of course not innocent which therefore limits this procedure to the fluorination of 2-pyridyl-bromobenzenes (Mu et al., 2014).

As highlighted with all examples commented above, intense research efforts to extend halogen exchange reactions to aryl halides led to the development of efficient processes, either catalytic or stoichiometric in metal. While some limitations still remain, this had a significant impact in chemical synthesis and the number of applications of these procedures is continuously increasing. Selected examples of synthetic applications of halogen exchanges in aryl halides that will be overviewed in the next section of this review article will further highlight their synthetic usefulness.

### Synthetic applications of halogen exchanges in aryl halides

#### For the *in situ* generation of reactive aryl iodides

**Figure 14:**
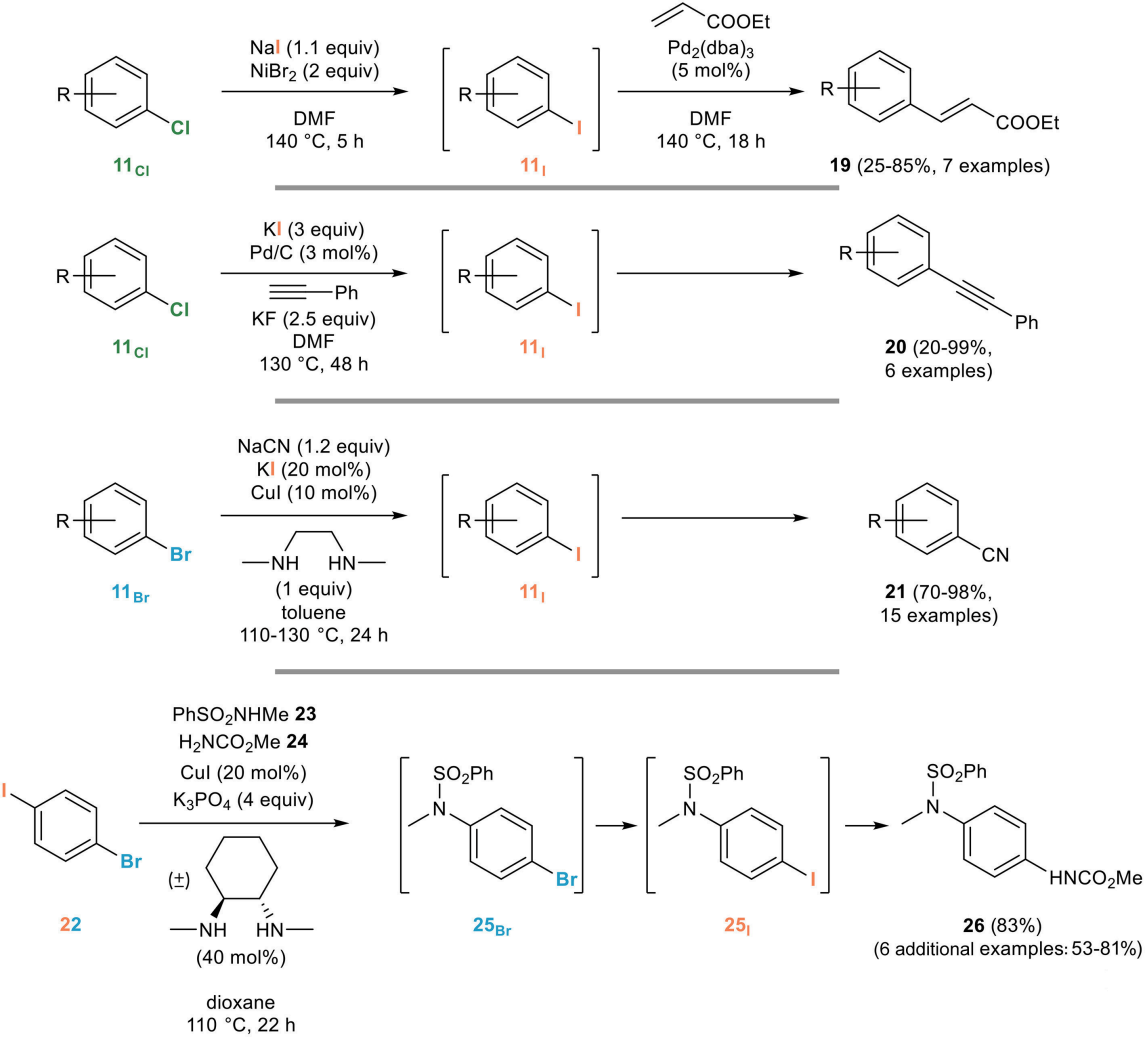
Representative applications of the aromatic Finkelstein reaction for the *in situ* generation of reactive aryl iodides.

The main application of the aromatic Finkelstein reaction is certainly the *in situ* generation of more reactive aryl iodides from the corresponding brominated or chlorinated derivatives. An early example can be found in the work of Bozell and Vogt on the use of aryl chlorides **11**_Cl_ for the Heck reaction (Bozell and Vogt, 1988): they indeed demonstrated that the difficulties associated with the use of these poorly reactive partners for the Heck reaction can be circumvented by their *in situ* nickel-mediated conversion to the corresponding and more reactive aryl iodides **11**_I_ which then undergo a smooth Heck coupling reaction with ethyl acrylate to **19** (Figure 14).

A closely related example relying on palladium catalysis was used for the *in situ* generation of aryl iodides from the corresponding chlorides, upon reaction with potassium iodide and palladium on carbon, the exact same heterogeneous catalyst being responsible for the subsequent Sonogashira coupling with phenylacetylene to provide the unsymmetrical diarylalkyne **20** (Thathagar and Rothenberg, 2006).

An iodide being released in such processes, the use of a stoichiometric amount of the iodinating agent is not strictly required, as evidenced by the Buchwald group who developed a remarkably efficient copper-catalyzed domino halogen exchange/cyanation reaction relying on the use of only 20 mol% of potassium iodide for the *in situ* conversion of aryl bromides **11**_Br_, poor substrates for the cyanation reaction, to their more reactive iodinated derivatives **11**_I_ that are rapidly cyanated to the corresponding benzonitriles **21** (Zanon et al., 2003).

An elegant application of the aromatic Finkelstein reaction was developed by the Willand group who noted that the copper-catalyzed cross-coupling of secondary amides with 1-bromo-4-iodobenzene mostly provided *N*-(4-iodophenyl)amides, resulting from an Ullmann/Finkelstein sequence, rather than the corresponding brominated derivatives (Toto et al., 2006a). Based on this observation, they developed a three component reaction from 1-bromo-4-iodobenzene **22** and two nitrogen nucleophiles with different reactivities toward Ullmann cross-coupling reactions, as exemplified in Figure 14. Copper-catalyzed cross-coupling involving the most acidic nitrogen nucleophile (sulfonamide **23** in Figure 14) followed by an aromatic Finkelstein reaction converting **25**_Br_ to **25**_I_ and subsequent cross-coupling involving the less reactive nucleophile (carbamate **24** in Figure 14) smoothly gave the dissymmetrical product **26** (Toto et al., 2006b). Other examples featuring an *in situ* copper-catalyzed aromatic Finkelstein reaction followed by *N*- (Jones et al., 2007) or *S*- (Carril et al., 2007) arylation have been reported for the synthesis of anilides or arylthioethers from poorly reactive aryl bromides that can be transformed into the corresponding more reactive aryl iodides prior to their cross-coupling. Finally, it ought to be mentioned that the efficiency of the copper-catalyzed aromatic Finkelstein reaction has made it an attractive tool in material sciences and in supramolecular chemistry, as evidenced by its use in the preparation of molecular chains (Vicente et al., 2005, 2008), oligothiophenes (Masuda et al., 2009) or molecular motors (Carella et al., 2008).

As mentioned in the introduction of this manuscript, the extension of the halogen exchange reaction to the introduction of radionuclides is especially relevant for medical imaging in which radiotracers embedded with labeled aryl fluorides (^18^F) and iodides (^123^I) are important. While the conditions reported to date for the fluorination of aryl halides do not allow for its extension to radiolabeling, the development of efficient processes for the iodination of aryl halides proceeding under mild enough conditions recently led to their use for the rapid synthesis of tracers for SPECT imaging. Advances in this area will be exemplified in the following paragraphs.

#### For radioiodination

SPECT imaging with radioiodinated probes has indeed found widespread applications in diagnostic imaging (^123^I) and biological research (^125^I). While the half-life of ^125^I (59.5 days) is long enough for most processes reported for the iodination of aryl halides to be extended to radioiodination, the considerably shorter half-life of ^123^I (13.2 h) renders the corresponding radioiodination much more challenging. The Sutherland group has been especially prolific in this area, clearly showing the potential of the halogen exchange reaction for radioiodination in a number of relevant cases, even if radionuclides were not used in most studies reported (Stevenson et al., 2007; Jobson et al., 2008; Cant et al., 2012).

**Figure 15:**
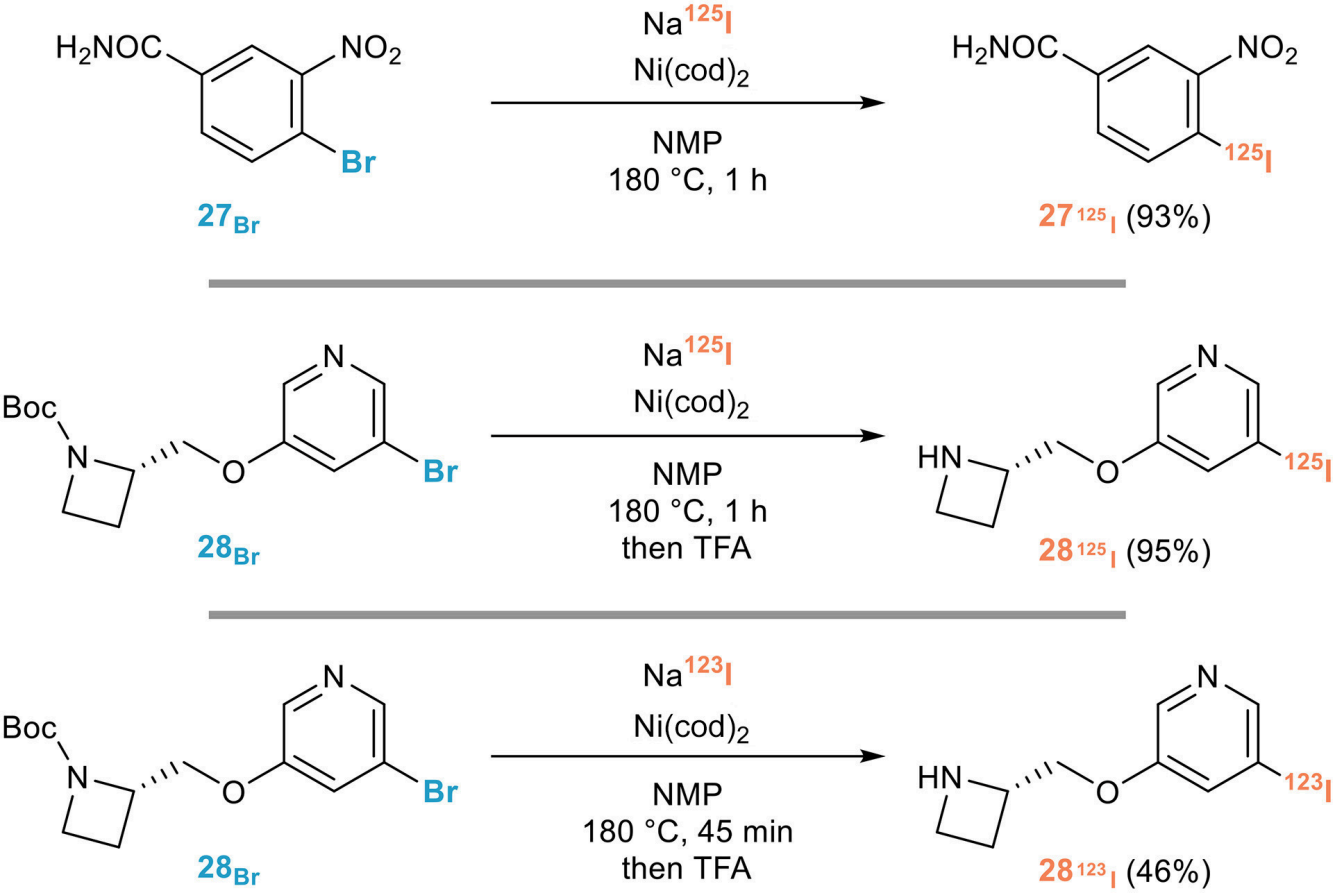
Representative applications of the aromatic Finkelstein reaction for radioiodination.

They notably reported in 2013 a remarkably efficient procedure applicable to the introduction of both ^123^I and ^125^I featuring short reaction times, a prerequisite for the use of ^123^I derivatives (Cant et al., 2013). Capitalizing on their previous adaptation of the Tagaki procedure, they indeed developed an efficient nickel-mediated radioiodination of aryl and heteroaryl bromides for the rapid synthesis of tracers for SPECT imaging. A screening of nickel catalysts revealed the high efficiency of Ni(cod)_2_ for the introduction of ^125^I from a series or (hetero)aryl bromides when the reaction was performed in NMP at 180°C for 1 h. The efficiency of this procedure could be unambiguously demonstrated by the synthesis of [^125^I]-iniparib **27**_125_I__, a drug candidate for oncology whose efficiency could however not be demonstrated in late-stage trials, or 5-[^125^I]-A85380 **28**_125_I__, a SPECT tracer used for imaging neuronal nicotinic acetylcholine receptors (nAChR) in humans, or its ^123^I analog **28**_123_I__, all these radioiodinated probes being obtained with remarkable efficiencies and excellent radiochemical yields (Figure 15).

Significant advances have been made toward the development of efficient and robust procedures for the catalysis of the halogen exchange in aryl halides with an array of metal complexes, even if some of these halogen exchanges still require harsh conditions and stoichiometric amounts of the metal complexes. The development of these procedures paved the way for their extension to halogenated alkenes which are at the core of the next section of this review article.

## Halogen exchange in alkenyl halides

As aryl halides, alkenyl halides are at the core of a range of natural and/or biologically relevant molecules and they are major starting materials for a variety of metal-catalyzed cross-coupling reactions. In such reactions, alkenyl iodides are usually better reaction partners than their lower homologs, while alkenyl chlorides are more commonly found in natural products and drugs than their brominated and iodinated counterparts. From a synthetic point of view, there are much more methods available for the stereoselective synthesis of alkenyl iodides—which can be readily obtained by iodolysis of vinylmetal species, Takai (Takai et al., 1986), or Stork-Zhao (Stork and Zhao, 1989) olefinations, hydroiodination of alkynes (Kawaguchi and Ogawa, 2010), iododesilylation (Chan and Fleming, 1979; Stamos et al., 1996; Arefolov et al., 2001; Ilardi et al., 2008), destannylation (Jung and Light, 1982; Darwish and Chong, 2012), or Hunsdiecker-type reactions (Das and Roy, 2002; Kulbitski et al., 2011)—than for the synthesis of chlorinated or brominated alkenes. Based on these considerations, being able to efficiently exchange one halogen atom by another one in a stereospecific manner is a fully relevant transformation for which there are now a set of efficient procedures available: they will be reviewed in this section.

### Vinylic finkelstein reactions

The vinylic Finkelstein reaction has been far less investigated compared to the aromatic one, despite its important potential, and the scope of most procedures reported to date has in general not been fully investigated.

**Figure 16:**
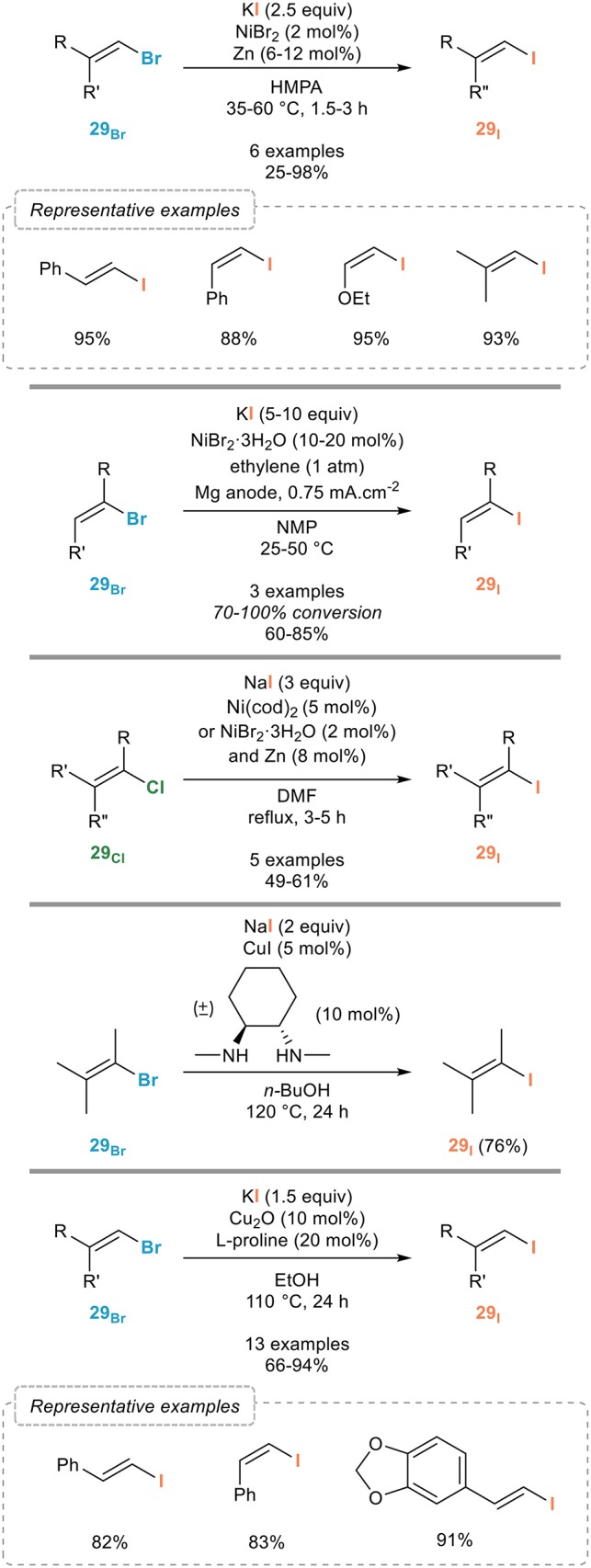
Vinylic Finkelstein reactions.

Most catalytic systems reported for this transformation were actually based on the ones developed for the halogen exchange in aryl halides which could be, in most cases, extended to the vinylic Finkelstein reaction. The most representative and significant ones are shown in Figure 16. The success met with nickel(0) complexes in the aromatic series prompted several groups to investigate the extension of the Finkelstein reaction to alkenyl halides. Early examples, which still represent an efficient procedure for the vinylic Finkelstein reaction, were reported by the Takagi group (Takagi et al., 1978a) who adapted their own procedure (Takagi et al., 1978b) to alkenyl bromides **29**_Br_ (Figure 16, top). Upon reaction with catalytic amounts of nickel(II) bromide and zinc with potassium iodide as the iodinating agent, various alkenyl bromides **29**_Br_ were shown to be smoothly transformed to their iodinated analogs **29**_I_ in fair to good yields and, importantly, with full conservation of the double bond geometry. Electro-generated nickel complexes stabilized by coordination to ethylene were also found to be efficient, although a full conversion was not observed in all cases (Meyer et al., 1986) (Figure 16, middle-top). Interestingly, and in sharp contrast with the limited efficiency of nickel catalysis for the transformation of aryl chlorides to their higher homologs, the Brandsma group (Hooijdonk et al., 1994) demonstrated their efficacy for the conversion of chlorinated alkenes **29**_Cl_ to the iodinated ones **29**_I_ provided that the reaction was performed either with Ni(cod)_2_ or a combination of NiBr_2_·3H_2_O and zinc in refluxing DMF (Figure 16, middle).

Copper(I) catalyst are equally efficient, if not superior, to nickel complexes: while early work from the Suzuki group relied on rather harsh conditions stoichiometric in copper (Suzuki et al., 1988), the introduction of chelating ligands for copper enabled the development of milder conditions for the vinylic Finkelstein reaction, as shown by the Buchwald group (Klapars and Buchwald, 2002) who demonstrated that the catalytic system developed for the aromatic Finkelstein reaction, based on a combination of sodium iodide, copper(I) iodide and (±)-*trans*-*N*,*N*′-dimethyl-1,2-cyclohexanediamine, was also efficient for the conversion of alkenyl bromides **29**_Br_ to the corresponding iodides **29**_I_ (Figure 16, middle-bottom). Although a single example was reported on their publication, the number of uses of this procedure clearly demonstrated its generality (Humphreys et al., 2006; Vaswani and Chamberlin, 2008). An alternative catalytic system, also resulting from an extension of an aromatic halogen exchange reaction, was recently reported by the Bao group (Feng et al., 2017): the combination of copper(I) oxide and proline reported for the conversion of aryl bromides to the corresponding iodides gave equally efficient results with alkenyl bromides **29**_Br_ that could be converted to their iodinated homologs **29**_I_ with high efficiency (Figure 16, bottom). As for photoinduced processes, they were shown to be efficient but non-stereospecific due to isomerization of the halogenated alkenes under UV irradiation, which clearly limits the significance of this approach in the vinylic series (Li et al., 2015).

### Vinylic retro-finkelstein reactions

**Figure 17:**
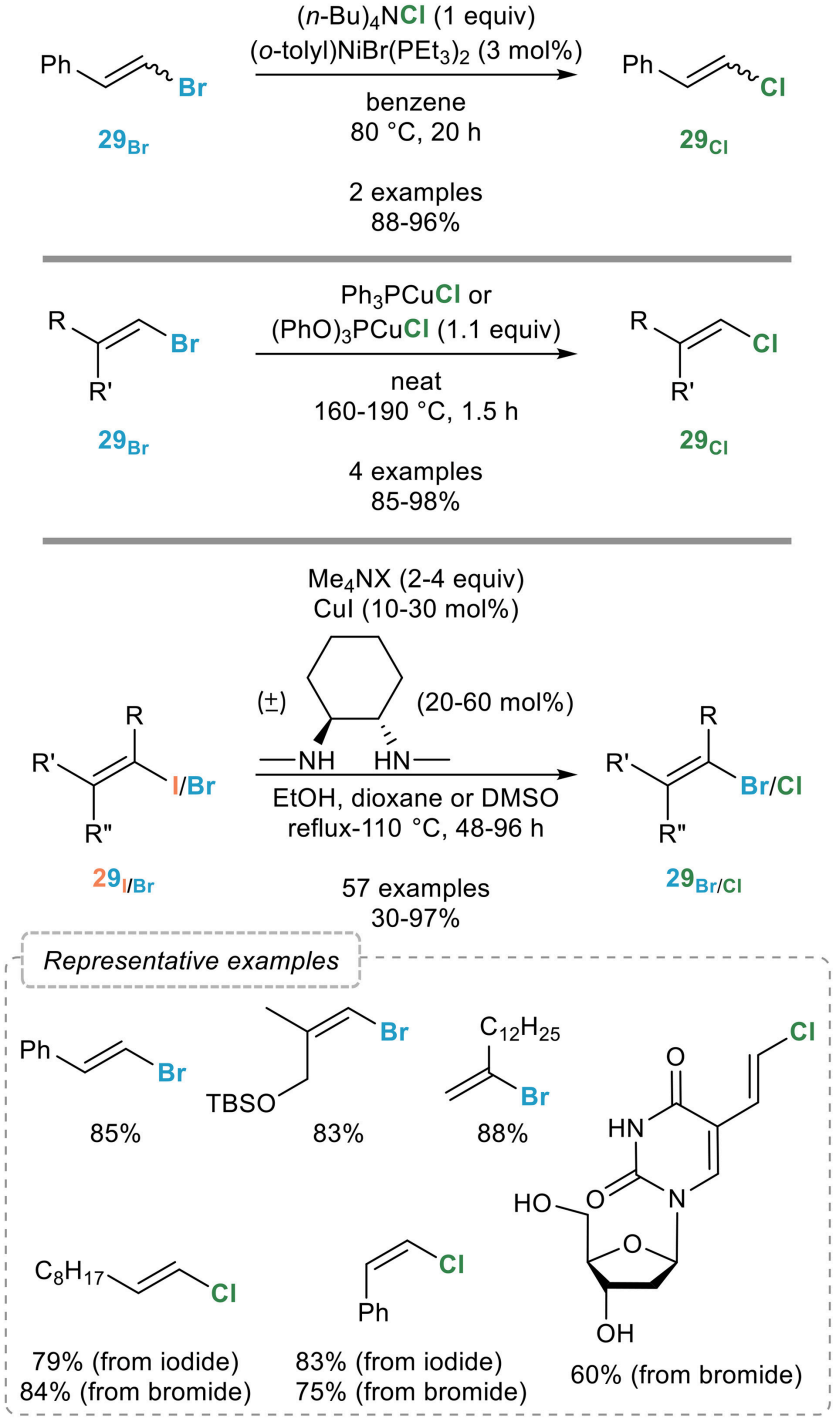
Vinylic retro-Finkelstein reactions.

As mentioned above, the main interest of the vinylic retro-Finkelstein lies in its potential to convert readily available alkenyl iodides into the corresponding chlorinated alkenes which are much more challenging to prepare in a stereoselective manner. Surprisingly, little attention has been paid to this interesting and useful transformation. Indeed, a meticulous literature analysis reveals that only two examples by the Kochi group have been reported on the use of nickel catalysis for the vinylic retro-Finkelstein reaction, based on the catalytic system they developed in the aromatic series (Figure 17, top): while this process also seem to be efficient for the conversion of brominated alkenes **29**_Br_ to the chlorinated ones **29**_Cl_, the scope of this process clearly would deserve further exploration (Tsou and Kochi, 1980).

At the same time, Axelrad (Axelrad et al., 1981a,b) demonstrated that phosphite- or phosphine- ligated copper(I) chloride complexes could mediate the transformation of alkenyl bromides to the corresponding chlorides, although under harsh conditions and using a stoichiometric amount of the copper complex (Figure 17, middle).

More recently, we reported a general procedure for the transformation of alkenyl iodides **29**_I_ and bromides **29**_Br_ to their lower homologs upon reaction with the corresponding tetramethylammonium halides and a combination of copper(I) iodide and (±)-*trans*-*N*,*N*′-dimethyl-1,2-cyclohexanediamine in ethanol at 110°C (Figure 17, bottom). The scope of this process was found to be general since complete conversions and good to excellent yields were obtained in most cases, substitution close to the reacting center however requiring higher catalytic loadings. The geometry of the starting alkene was preserved in all cases and key features of this vinylic retro-Finkelstein reaction are its broad applicability and the mild reaction conditions compatible with a range of highly functionalized substrates. The potential of this vinylic halogen exchange reaction in total synthesis and medicinal chemistry was demonstrated by its successful use for the synthesis of the C1-C9 fragment of laingolide B and for the late-stage modification of drug-like molecules (Nitelet and Evano, 2016; Nitelet et al., 2016).

## Conclusions and outlook

As overviewed with all examples highlighted in this review article, significant advances have been reported toward the development of efficient and broadly applicable procedures for the halogen exchange in aryl and alkenyl halides. Some of these procedures have been readily adopted by many chemists and they are now part of the useful and reliable tools that can be used for chemical synthesis.

When looking at the conclusion of the previous review on the halogen exchange in aryl halides published by Sheppard in 2009 (Sheppard, 2009), major advances have been made in the last decade. The fluorination of aryl halides is indeed now possible and has been implemented in a number of processes, even if procedures catalytic in copper are still to be developed and if its extension to alkenyl halides is still elusive while general procedures have been reported for the vinylic (retro-)Finkelstein reaction.

Challenges still remain however. One of the most important ones is certainly the development of efficient catalytic processes for the aromatic Finkelstein reaction from aryl chlorides under mild conditions: recent reports from the Ma group on the copper-catalyzed activation of aryl chlorides (Zhou et al., 2015; Fan et al., 2016) open new perspectives in this area and should enable the design and development of efficient processes catalytic in copper for this transformation. Another main challenge is the development of procedures amenable to the use of radionuclides: in most cases, the time required for the halogen exchange to proceeds is still way too important for radionuclides with short half-lives to be used in such processes. As with most transformations, and as nicely evidenced with recent advances on the palladium-catalyzed fluorination of aryl halides, a better understanding of the reaction mechanisms will be the key toward the development of more efficient processes for the halogen exchange in aryl and vinyl halides.

## Author contributions

AN: Collected most publications related to this review article, sorted them out and wrote the first draft of the manuscript; PT and DD: Completed the literature analysis and extracted and compiled all data from the publications; GE: Wrote the manuscript based on the literature analysis and on the first draft.

### Conflict of interest statement

The authors declare that the research was conducted in the absence of any commercial or financial relationships that could be construed as a potential conflict of interest.
